# Investigation of cellular uptake mechanism of functionalised gold nanoparticles into breast cancer using SERS†

**DOI:** 10.1039/d0sc01255f

**Published:** 2020-05-27

**Authors:** Anastasia Kapara, Valerie Brunton, Duncan Graham, Karen Faulds

**Affiliations:** Pure and Applied Chemistry, Technology and Innovation Centre, University of Strathclyde 99 George Street Glasgow Scotland G1 1RD UK karen.faulds@strath.ac.uk; Edinburgh Cancer Research UK Centre, University of Edinburgh Crewe Road South Edinburgh Scotland EH4 2XU UK

## Abstract

Gold nanoparticles (AuNPs) are widely used in various applications such as cancer imaging and drug delivery. The functionalisation of AuNPs has been shown to affect their cellular internalisation, accumulation and targeting efficiency. The mechanism of cellular uptake of functionalised AuNPs by different cancer cells is not well understood. Therefore, a detailed understanding of the molecular processes is necessary to improve AuNPs for their selective uptake and fate in specific cellular systems. This knowledge can greatly help in designing nanotags with higher cellular uptake for more selective and specific targeting capabilities with less off-target effects. Here, we demonstrate for the first time a straightforward and non-destructive 3D surface enhanced Raman spectroscopy (SERS) imaging approach to track the cellular uptake and localisation of AuNPs functionalised with an anti-ERα (estrogen receptor alpha) antibody in MCF-7 ERα-positive human breast cancer cells under different conditions including temperature and dynamin inhibition. 3D SERS enabled information rich monitoring of the intracellular internalisation of the SERS nanotags. It was found that ERα-AuNPs were internalised by MCF-7 cells in a temperature-dependent manner suggesting an active endocytosis-dependent mechanism. 3D SERS cell mapping also indicated that the nanotags entered MCF-7 cells using dynamin dependent endocytosis, since dynamin inhibition resulted in the SERS signal being obtained from, or close to, the cell surface rather than inside the cells. Finally, ERα-AuNPs were found to enter MCF-7 cells using an ERα receptor-mediated endocytosis process. This study addresses the role of functionalisation of SERS nanotags in biological environments and highlights the benefits of using 3D SERS for the investigation of cellular uptake processes.

## Abstract 2

Use of sensitive, non-destructive and straightforward 3D SERS for investigating the cellular uptake processes of functionalised nanotags in entire cell volume.

## Graphical abstract

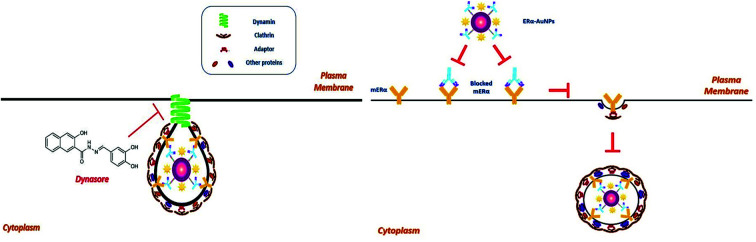

## Introduction

Gold nanoparticles (AuNPs) have been extensively investigated as tools for sensing and tracking of biomedically important cellular markers in a broad range of applications including *in vitro*^1^ and *in vivo*^2,3^ imaging. The effective design of AuNPs, for dynamic cell imaging and biocompatibility, requires careful consideration of their fundamental cellular uptake interactions within living systems. In general, these investigations include different studies to determine the amount and location of internalised nanotags (AuNPs + biomolecule + reporter) and they are usually conducted in conjunction with viability and inhibition studies that block individual cellular uptake mechanisms.^4^

The intracellular uptake and fate of AuNPs is dependent on different factors, such as their physicochemical characteristics^5–7^ and the experimental procedures, including incubation time and AuNP concentration.^8,9^ The functionalisation of AuNPs with targeting biomolecules greatly affects their trafficking behaviour and their cellular localisation.^10^ The binding of biomolecule functionalised AuNPs to their cellular targets increases the accumulation of the AuNPs in the cell and minimises exocytosis processes.^9,11,12^ Antibody-conjugated nanoparticles have been shown to have increased cellular uptake^13^ due to the presence of the antibodies on the nanoparticle surface affecting the nanoparticle–cell interactions, leading to enhanced signals and long-term tracking of antigen expression in the cell.^14^

Different uptake mechanisms exist for the cellular internalisation of AuNPs including phagocytosis, micropinocytosis, clathrin- and caveolae-dependent and clathrin- and caveolae-independent endocytosis.^15^ Endocytosis involves the formation of new intracellular membrane-enclosed vesicles from the cell membrane with a concomitant internalisation of the cargo along with other proteins, lipids and extracellular fluids.^15^ It has been shown that the energy-dependent endocytosis pathways, rather than passive diffusion, are the main mechanisms that cell lines use for nanoparticle uptake.^16,17^ Specifically, receptor-mediated endocytosis (RME) is known to be one of the main uptake pathways for AuNPs. For RME, the biomolecules attached to the AuNP surface bind to the extracellular surface of the plasma membrane receptor and membrane fusion is induced. This membrane enfolding leads to the formation of an endosome, which allows the cell to carry the cargo into the cytosolic region.^18,19^ RME involves participation of other proteins, such as clathrin or caveolae, for the cellular internalisation mechanisms.^20,21^

Traditionally, transmission electron microscopy (TEM)^22^ and fluorescence microscopy^23,24^ have been used for investigating cellular uptake and localisation of nanoparticles in cells. However, TEM is a destructive and expensive technique that requires microtoming of the cells with long and complicated sample preparation. On the other hand, immunostaining using primary and secondary antibodies for subsequent fluorescence imaging requires the cell membrane to be disrupted to allow the antibodies access to their targets within the cell. This usually requires permeabilization, *i.e.* the creation of holes in the cell membrane. In addition, the fluorophores that are required to stain cells for fluorescence imaging are prone to photobleaching, making three dimensional (3D) imaging challenging since bleaching can compromise definition of 3D structures leading to false results.^25^ Additionally, fluorescence generates broad emission bands that makes the detection of multiple components within the same sample challenging.^26^

Therefore, high-resolution optical imaging has gained increasing importance, providing clear evidence of nanotag cellular uptake and localisation in a non-destructive fashion. Surface enhanced Raman spectroscopy (SERS) is a non-destructive method that can study the interactions of nanotags with biological environments with various advantages, such as high sensitivity, selectivity and multiplexing capacities, without the need for fluorogenic staining.^1,27^ Recently, nanoparticle-based SERS approaches have been conducted to map the intracellular distribution of different molecules in fixed^28–31^ and live cells^32–34^ allowing different cellular functions and compartments to be monitored. The addition of a Raman reporter to the surface of AuNPs gives a characteristic signal that is distinctive from the intrinsic Raman signal from the cell components. This allows visualisation of the AuNPs localisation with high multiplexing capabilities and photostability.

In this study, we introduce for the first time the use of non-destructive 3D SERS imaging for the investigation of the cellular uptake mechanisms of AuNPs functionalised with an anti-ERα (estrogen receptor alpha) antibody and BPE (1,2-bis(4-pyridyl)ethylene) Raman reporter (ERα-AuNPs), in breast cancer cells under different endocytosis pathway inhibition conditions. Additional novelty comes from the ability to investigate the cellular uptake and localisation of SERS nanotags in the entire volume of the cell. The collected data were processed and analysed as one data set making SERS a quick and affordable technique, in contrast to TEM that requires laborious sample preparation with potential artefacts as well as being destructive. Additionally, in the protocol described here, the antibody functionalised nanoparticles are only incubated with the cells for a short time making the imaging much faster compared to fluorescent staining that requires two antibodies, a primary antibody and then a fluorescently labelled secondary antibody. The ability to investigate the cellular uptake and cellular accumulation of SERS nanotags using a sensitive and non-destructive technique is of crucial importance for the validation of AuNPs as an important tool in optical medical imaging.

## Results and discussion

### Nanoparticle synthesis and characterisation of ERα-AuNPs

AuNPs were synthesised using a standard citrate reduction method^35^ and characterised using extinction spectroscopy, dynamic light scattering (DLS), zeta potential analysis and scanning electron microscopy (SEM). The results revealed that the AuNPs had a spherical shape and were 40–50 nm in diameter (ESI, Fig. S1†). Anti-ERα antibodies were attached to the gold surface *via* carbodiimide crosslinking chemistry, which created an amide bond between the carboxylic acid of a polyethylene glycol molecule (HS-PEG5000-COOH) and an amine group on the antibody^36^ (ESI, Fig. S2†). The HS-PEG5000-COOH was used to prevent nonspecific interactions between the functionalised nanotags and other cellular components. No significant decrease in SERS signal was observed after the functionalisation of bare AuNPs (ESI, Fig. S3†). The coupling chemistry was achieved after the attachment of the BPE Raman reporter, to the AuNP surface *via* the pyridyl nitrogens.^37^ The successful bioconjugation of ERα-AuNP nanotags was characterised using extinction spectroscopy, DLS, agarose electrophoresis and a lateral flow immunosorbent assay (ESI, Fig. S4†). It can be seen from the extinction spectra in Fig. S4A,† that the ERα-AuNP nanotags do not aggregate indicating that they were stable after functionalisation. DLS confirmed the successful antibody functionalisation since the hydrodynamic diameter of the nanotags increased from 73 ± 1.0 nm to 80 ± 1.6 nm at pH 7.0 after the bioconjugation (ESI, Fig. S4B†). Moreover, a decrease in zeta potential was observed after the addition of the positively charged anti-ERα antibody (from −56 ± 0.7 mV to −52 ± 1.1 mV) confirming the successful attachment of the antibody to AuNPs surface (ESI, Fig. S4C†). Additionally, agarose electrophoresis verified that the PEG5000-AuNPs travelled further than the ERα-AuNPs, suggesting that ERα-AuNP nanotags were of a larger size (ESI, Fig. S4D†) which is consistent with the DLS data. Finally, a lateral flow immunosorbent assay showed that the antibodies on the surface of the nanotags were active since a spot from ERα-AuNPs was observed only when the matching secondary IgG antibody for ERα was applied on the strip (ESI, Fig. S4E and S5†). The nanotags did not show any aggregation in the extinction spectra, indicating the AuNPs were stable after the addition of the antibody and Raman reporter to the metal surface. The quantification of the ERα antibody conjugated to AuNPs surface was estimated using a bicinchoninic acid assay (BCA). The results showed that the average number of ERα antibody molecules adsorbed per AuNP was 64 ± 5.9 (ESI, Fig. S6†). The nanotags were also analysed using SERS on a Snowy Range CBEx 2.0 handheld Raman spectrometer equipped with a 638 nm laser with a maximum laser power of 40 mW. The results showed that the nanotags gave a strong and characteristic SERS signal from the BPE Raman reporter (ESI, Fig. S7†). Finally, although ERα-AuNPs were prepared fresh before their addition to the cells, they showed sufficient stability in both H_2_O and cell media solutions (ESI, Fig. S8†). Therefore, the AuNPs were successfully functionalised, stable and could be further used in cell studies.

### Characterisation of breast cancer cells

The MCF-7 breast cancer cell line is known to overexpress the ERα biomarker. To confirm the expression of ERα, a western blot was performed which showed that ERα was present in the MCF-7 cells (ESI, Fig. S9†). Therefore, the MCF-7 cells were used to study the uptake of the ERα-AuNP nanotags.

### Cytotoxicity evaluation of ERα-AuNP nanotags in MCF-7 cells

**Fig. 1:**
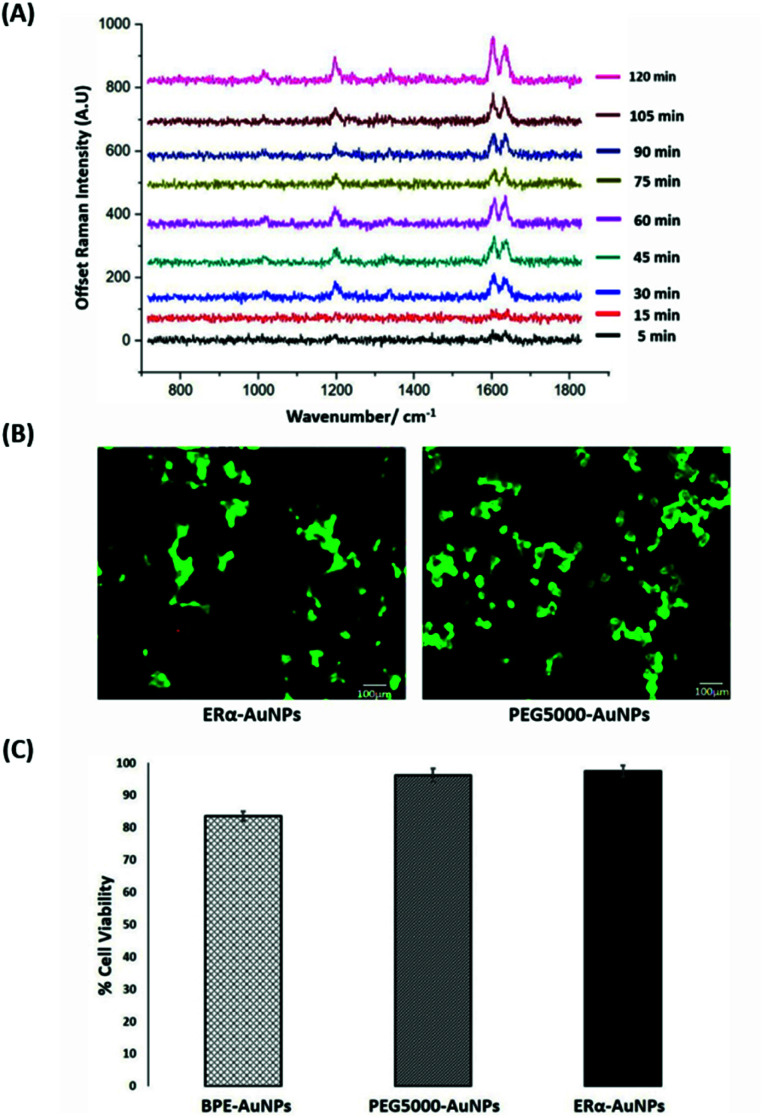
ERα-AuNPs incubated in MCF-7 cells showed strong SERS signal with no detectable cell toxicity. (A) Average SERS signal from ERα-AuNPs in MCF-7 cells under different nanotag incubation times ranging from 5 min to 120 min. SERS spectra were collected in fixed MCF-7 cells using a Renishaw InVia system combined with edge Streamline HR high confocality mode at 1 μm resolution in the *X* and *Y* directions. A 50× magnification NIR APO Nikon water immersion objective with a 0.75 NA was used on the samples at a laser power of 1.2 mW (10% power) at the sample, from a HeNe 633 nm excitation source with a 0.1 s acquisition time per point, and a 1200 L mm^−1^ grating in high confocality mode. Windows-based Raman Environment (WiRE™ – Renishaw plc) 4.4 software package was used to pre-process the data for cosmic ray removal and baseline subtraction. (B) Cell viability assay of MCF-7 cells treated with 60 pM ERα-AuNPs (left) and 60 pM PEG5000-AuNPs (nanotags without ERα antibody functionalisation) (right) for 48 h using live/dead staining with Calcein AM and EthD Br-1 assay. Viable cells appear as green (Calcein AM), while non-viable cells appear as red (EthD Br-1). Scale bar 100 μm. (C) Cell viability using trypan blue assay for MCF-7 cells treated with 60 pM BPE-AuNPs, 60 pM PEG5000-AuNPs or 60 pM ERα-AuNPs for 48 h. The average of ten samples from three independent biological replicates is shown. Error bars presented as mean ± standard deviation (SD).

SERS was utilised first to investigate the accumulation of ERα-AuNP nanotags in MCF-7 breast cancer cells under different cellular incubation conditions. Specifically, MCF-7 cells were incubated with the nanotags at different concentrations, incubation times and temperatures. The results showed that the MCF-7 cells had high nanotag accumulation and a strong SERS signal after 120 min incubation with the ERα-AuNP nanotags (Fig. 1A). The SERS signal at 75 min incubation was slightly lower than at 60 min and 90 min due to biological variation between the different fixed cells. Therefore, a 120 min incubation time was used in subsequent experiments. The bright-field images showed that the cells treated with 60 pM ERα-AuNPs were adherent, while higher nanotag concentrations caused changes to MCF-7 cell morphology in comparison to the untreated cells (data not shown). To further test the effect of ERα-AuNPs on the viability of MCF-7 cells, live/dead staining with green-fluorescent Calcein-AM and red-fluorescent ethidium homodimer-1 (EthD Br-1) was performed after incubation of ERα-AuNPs in MCF-7 cells for 48 h. The viability studies verified that ERα-AuNPs showed good biocompatibility without any obvious toxic effects on the MCF-7 cells over the culture period since a significant green colour corresponding to viable cells (Calcein AM) was present in contrast to a red colour, corresponding to non-viable cells (EthD Br-1) (Fig. 1B). Further cell viability studies were carried out using a trypan blue cell viability assay. The results showed that MCF-7 cells treated with AuNPs, coated with only the BPE Raman reporter, exhibited approximately 85% viability in contrast to the PEGylated BPE-AuNPs (96% cell viability). MCF-7 cells had the highest cell viability (97% cell viability) when they were incubated with ERα-AuNP nanotags (Fig. 1C). These results indicated that 60 pM of ERα-AuNPs incubated with MCF-7 for 48 h did not cause any cell toxicity indicating good biocompatibility of the nanotags.

### Calculation of relative SERS response value in MCF-7 cells

**Fig. 2:**
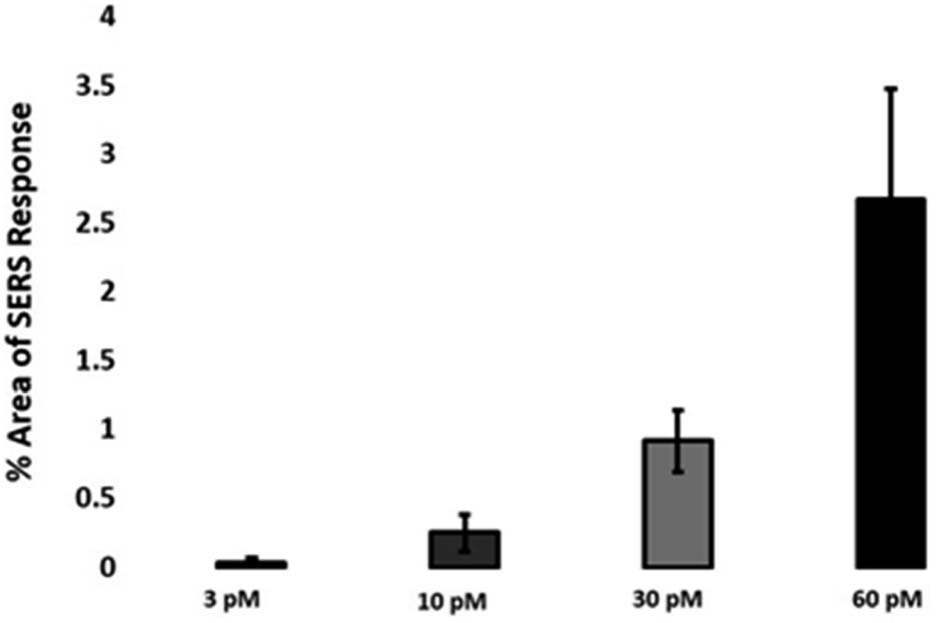
Calculation of relative SERS response value under different ERα-AuNPs concentrations. The calculations were carried out using WiRE™ – Renishaw plc 4.4 and Fiji image processing package. The average of ten samples from three independent biological replicates is shown. Error bars presented as mean ± SD.

The quantitative estimation of the number of SERS nanotags inside cells is a very challenging process since the exact number of AuNPs in each aggregate cannot be simply identified from the SERS signal obtained for each image. Therefore, we developed a method to calculate the number of pixels that corresponded to ERα-AuNPs in MCF-7 cells, based on the location of the SERS response, *versus* the total cell area mapped. This approach gives an estimation of the percentage of SERS responsive pixels per cell and provides an indication of the relative value for the uptake of nanotags per condition as the SERS signal will increase with the number of nanotags uptaken. It should be noted that this is not a direct quantification of the total number of nanotags in the cells. In this way, we can obtain a relative assessment of nanotag uptake using a direct, rapid and non-destructive optical approach averaged across multiple cells (10 per condition investigated). This is of huge advantage compared to other techniques used for the analysis of nanotags in cells such as TEM which is highly destructive, time consuming and expensive and it would be very challenging to measure enough cells to get a meaningful average statistical value per condition. Here, the exposure of MCF-7 cells to different ERα-AuNPs concentrations (3–60 pM) for 2 h showed that 60 pM is an effective concentration to produce a high SERS response per cell (Fig. 2) without affecting the viability of MCF-7 cells (Fig. 1B and C). Therefore, 60 pM ERα-AuNPs was used in subsequent experiments.

### ERα-AuNPs enter MCF-7 cells using a temperature dependent process

**Fig. 3:**
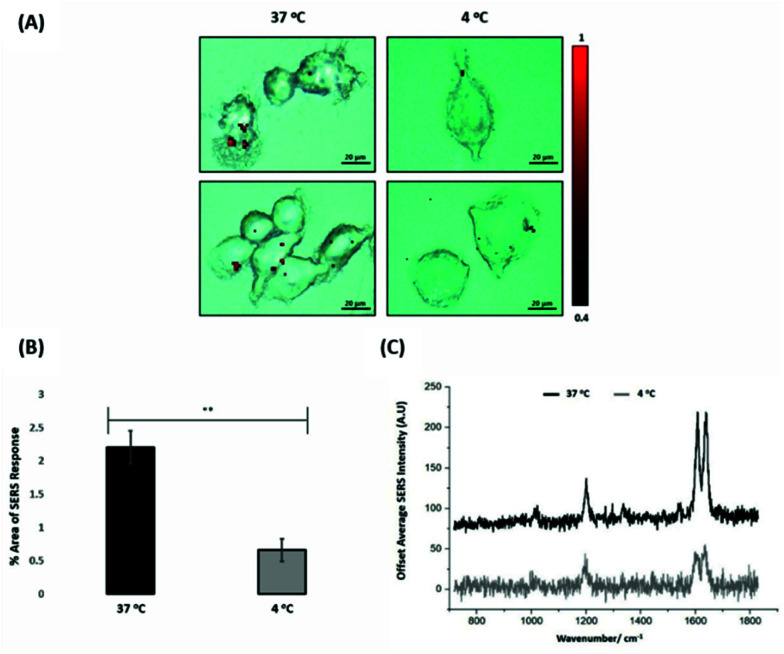
ERα-AuNPs enter MCF-7 cells in a temperature dependent manner (A) SERS map of MCF-7 cells treated with ERα-AuNPs (60 pM) for 2 h at 37 °C (left) and 4 °C (right). The images were generated using a Renishaw InVia Raman microscope with 50× magnification NIR APO Nikon water immersion objective with a 0.75 NA and 1.2 mW laser power (10% power) from a HeNe 633 nm excitation source with step size *y*,*x* 1.0 μm, 0.1 s acquisition time and a 1200 L mm^−1^ grating in high confocality mode. The red false colour images, representing ERα-AuNPs, were generated using WiRE™ Renishaw plc 4.4 software and direct classical least square analysis (DCLS) based on a BPE Raman reporter spectrum. Results are representative of 3 independent experiments (SERS mapping of 10 cells in each experiment). Scale bars = 20 μm. (B) Percentage of relative SERS response value in MCF-7 cells incubated with ERα-AuNPs (60 pM for 2 h) at 37 °C (black) and 4 °C (grey). The area was calculated using the Fiji image processing package by calculating the red pixel number, corresponding to ERα-AuNPs, and the mapped cell area. The average of ten samples from three independent biological replicates is shown. Error bars presented as mean ± SD. *Significant difference (*p* < 0.05) in a Student's *t*-test. (C) Representative average SERS spectra of MCF-7 cells incubated with ERα-AuNPs (60 pM for 2 h) at 37 °C (black) and 4 °C (grey) calculated from 10 cells of 3 independent experiments.

For the evaluation of the ERα-AuNPs uptake mechanisms in MCF-7 cells, a low-temperature assay was performed to investigate whether the nanotags were using an energy-dependent mechanism to enter the cells. In this experimental approach, MCF-7 cells were incubated with ERα-AuNPs (60 pM) at either low temperature (4 °C) or at normal incubation temperature (37 °C) for 2 h. The cells incubated at 4 °C were observed to retain their cell viability as they continued to grow in the flask and retained their cell morphology and no dead cells were detected. Previous studies have shown that if the nanoparticles enter cells *via* endocytosis, then a decrease in their cellular uptake is observed when the temperature is lower.^15,38^ Here, it was observed that there was a lower level of ERα-AuNP nanotag accumulation into MCF-7 cells at 4 °C, compared to the cells treated with the nanotags at 37 °C (Fig. 3A). Specifically, there was around a threefold decrease in the percentage of SERS response in MCF-7 cells treated with ERα-AuNPs at 4 °C in comparison to 37 °C (Fig. 3B). Additionally, there was a lower average SERS signal in the MCF-7 cells treated with ERα-AuNPs at 4 °C compared to 37 °C (Fig. 3C). The decreased internalisation of ERα-AuNPs at 4 °C demonstrated that their cellular uptake is an energy- and temperature-dependent process. The low signal observed at 4 °C was likely due to passive diffusion of a significantly lower number of nanotags into MCF-7 cells. This implies that the ERα-AuNPs interacted with the MCF-7 cells by non-passive diffusion transport since they require energy for their internalisation.

### ERα-AuNPs enter MCF-7 cells using dynamin dependent endocytosis

**Fig. 4:**
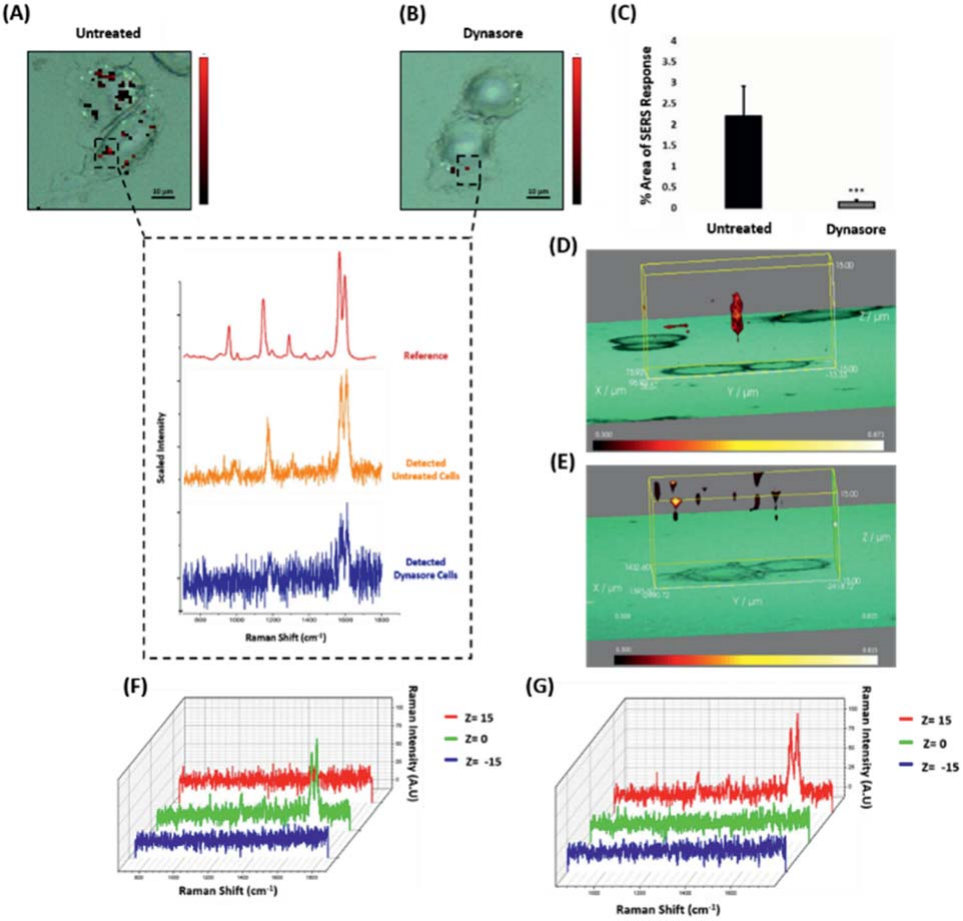
ERα-AuNPs use dynamin for their cellular uptake in MCF-7 cells. (A) False colour SERS map images for MCF-7 cells incubated with only ERα-AuNP nanotags (60 pM, 2 h) or (B) with ERα-AuNP nanotags (60 pM, 2 h) and dynasore (80 μM, 30 min). The inset (dashed box) shows the average SERS spectra from untreated (orange) and dynasore treated (blue) cells, stacked with BPE Raman reporter reference spectrum (attached on ERα-AuNPs) (red). The images were generated using a Renishaw InVia Raman microscope with 50× magnification NIR APO Nikon water immersion objective with a 0.75 NA and 1.2 mW laser power (10% power) from a HeNe 633 nm excitation source with step size *y*,*x* 1.0 μm, 0.1 s acquisition time and a 1200 L mm^−1^ grating in high confocality mode. Scale bar = 10 μm. (C) Calculation of relative SERS response value for MCF-7 cells incubated with ERα-AuNPs (60 pM, 2 h) or with dynasore (80 μM, 30 min) and ERα-AuNP nanotags (60 pM, 2 h). The calculations were carried out using WiRE™ – Renishaw plc 4.4 and Fiji image processing package. The average from three independent biological replicates is shown. Error bars presented as mean ± SD. (D) 3D SERS map from MCF-7 treated with only ERα-AuNPs (60 pM, 2 h) or (E) with dynasore (80 μM, 30 min) and ERα-AuNP nanotags (60 pM, 2 h). 3D SERS images were generated using the same conditions as those stated above for 2D mapping (Fig. 4A and B) except a step size of *z* = 3.0 was also employed. (F) 3D Raman mapping waterfall plot of average SERS spectra at different *z*-axis points, *z* = 15 μm (red), *z* = 0 μm (green) and *z* = −15 μm (blue) from MCF-7 treated with only ERα-AuNPs (60 pM, 2 h) or (G) with dynasore (80 μM, 30 min) and ERα-AuNP nanotags (60 pM, 2 h).

Nanotags functionalised with biomolecules can be internalised by cells using different pathways such as micropinocytosis, clathrin- or caveolin-mediated endocytosis. Typically, most of the nanotags are uptaken into cells by endocytosis after binding to membrane proteins.^39,40^ Clathrin is one of the main proteins that play a major role in the endocytosis pathway. Specifically, clathrin molecules self-assemble together to form a spherical coated vesicle, known as a clathrin coated vesicle (CCV). CCVs mediate the transport of cargo from the cell membrane inside the cell and between organelles.^41,42^ The formed CCV detaches from the membrane using dynamin, an intracellular GTPase protein that cleaves the neck of the vesicles being formed during endocytosis.^43,44^ Marczell *et al.* have shown that the ERα pathway is linked to dynamin-dependent receptor endocytosis in MCF-7 cells.^45^ Specifically, immunoelectron microscopy imaging showed that membrane bound ERα undergoes ligand-mediated receptor internalisation *via* a dynamin-dependent pathway.^45^ To demonstrate whether dynamin plays a role in ERα-AuNPs cellular uptake, MCF-7 cells were treated with dynasore, a GTPase inhibitor of dynamin.^46^ The use of dynasore aimed to inform whether the inhibition of dynamin would disrupt the internalisation of ERα-AuNPs. Therefore, MCF-7 cells were pre-treated with dynasore (80 μM for 30 min), the media was then removed, and fresh media was added to the cells before ERα-AuNPs (60 pM for 2 h) incubation. The 2D SERS mapping showed that the untreated MCF-7 cells had the highest SERS signals from the interior of the cells (Fig. 4A). In contrast, the dynasore treated cells showed reduced uptake of ERα-AuNPs where the highest signals were obtained from the cell surface (Fig. 4B). The lower accumulation of the nanotags in the dynasore treated cells was also verified from the observed significant reduction of SERS response (from 2.2% ± 0.7 to 0.2% ± 0.1) confirming that the dynamin-dependent pathway played an important role in the uptake of ERα-AuNPs (Fig. 4C). To further investigate the localisation of the nanotags, 3D SERS cell mapping was performed using a 0.1 s accumulation time per spectrum and 1.2 mW laser power with a 633 nm laser excitation source. Each cell map was obtained with a 1 μm lateral resolution in *x* and *y* directions and 3 μm in the *z*-direction (30 μm overall depth). The acquisition time for each 3D cell map was approximately 2 hours, depending on the shape and size of each cell. The mapping was performed in fixed cells to avoid the possibility of cell or organelle movement during the scanning acquisition time. The average SERS spectra per *z*-slice of the map showed a strong SERS signal within the cell in the untreated MCF-7 cells (Fig. 4D and F). The top view of the cell mapping also confirmed that the signal was generated within the cell area rather than the junction of the two cells (ESI, Fig. S10†). Most importantly, it confirmed that dynamin inhibition led to a significant reduction in the SERS signal throughout MCF-7 cells with the SERS signal seeming to be localised to the cell surface (Fig. 4E and G). The exact points in the cells that were used for the generation of SERS spectra throughout the *z*-axis are shown in ESI, Fig. S11.† Additionally, ESI Fig. S12† represents the spectra from each 3 μm step size of the *z*-stack in the whole volume map. 3D SERS imaging suggested that dynasore disrupted the formation of endocytic vesicles which led to ERα-AuNPs being trapped on the cell surface and not being internalised.

### ERα-AuNPs enter MCF-7 cells using membrane ERα

**Fig. 5:**
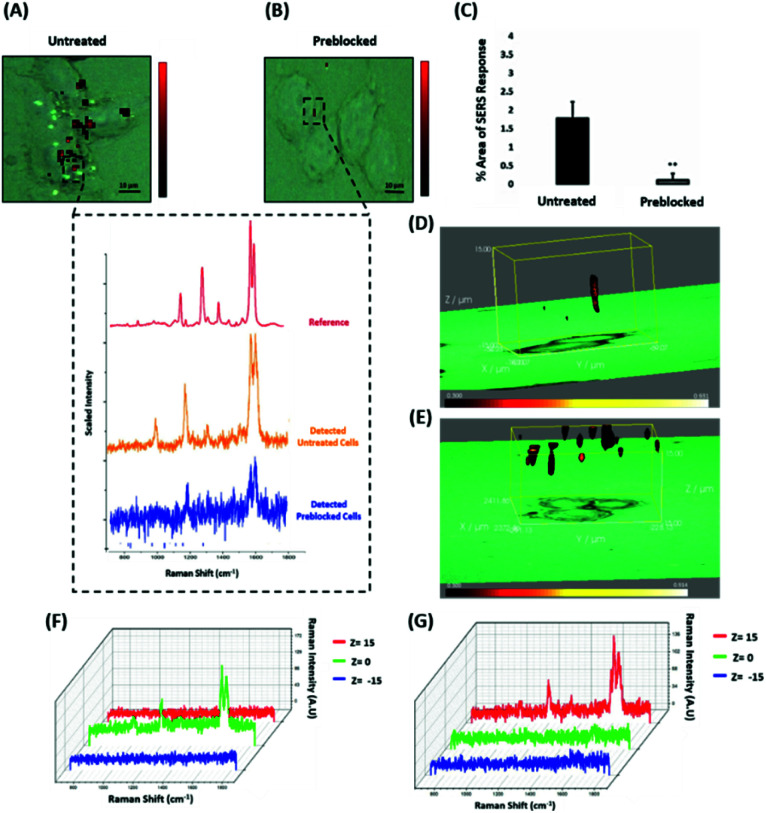
ERα-AuNPs use mERα for their cellular uptake in MCF-7 cells. (A) False colour SERS map images for MCF-7 cells incubated with ERα-AuNPs (60 pM, 2 h) or (B) pre-blocked with free anti-ERα antibody (10 μg mL^−1^, 1 h) and then treated with ERα-AuNPs (60 pM, 2 h). The inset (dashed box) shows the average SERS spectra from untreated (orange) and pre-blocked with free anti-ERα antibody (blue) cells stacked with reference spectrum from nanotags (red). The images were generated using a Renishaw InVia Raman microscope with 50× magnification NIR APO Nikon water immersion objective with a 0.75 NA and 1.2 mW laser power (10% power) from a HeNe 633 nm excitation source with step size *y*,*x* 1.0 μm, 0.1 s acquisition time and a 1200 L mm^−1^ grating in high confocality mode. Scale bar = 10 μm. (C) Calculation of relative SERS response value for MCF-7 cells incubated with ERα-AuNPs (60 pM, 2 h) or pre-blocked with free anti-ERα antibody (10 μg mL^−1^, 1 h) and then treated with ERα-AuNPs (60 pM, 2 h). The calculations were carried out using WiRE™ – Renishaw plc 4.4 and Fiji image processing package. The average from three independent biological replicates is shown. Error bars presented as mean ± SD. (D) 3D SERS map from MCF-7 cells treated with ERα-AuNPs (60 pM, 2 h) and (E) MCF-7 cells pre-blocked with free anti-ERα antibody (10 μg mL^−1^, 1 h) and then treated with ERα-AuNPs (60 pM, 2 h). 3D SERS images were generated using the same conditions as those stated above for 2D mapping (Fig. 5A and B) except a step size of *z* = 3.0 was also employed. (F) 3D Raman mapping waterfall plot of average SERS spectra into different *z*-axis points, *z* = 15 μm (red), *z* = 0 μm (green) and *z* = −15 μm (blue) from MCF-7 cells treated with only ERα-AuNPs (60 pM, 2 h) or (G) with free anti-ERα antibody (10 μg mL^−1^, 1 h) and then treated with ERα-AuNPs (60 pM, 2 h).

Since the results showed that ERα-AuNPs appeared to use dynamin for their internalisation in MCF-7 cells, another study was conducted to investigate if the nanotag uptake was dependent on receptor-mediated endocytosis. Receptor-mediated endocytosis is a process of specific recognition in which the cells internalise nanotags through binding with plasma membrane proteins specific to the molecules being internalised.^47,48^ The cell membrane region, that contains the receptor–ligand complex, then undergoes endocytosis using a transport vesicle. The rate at which the cargo is internalised is related to the amount of its corresponding receptor on the cell surface. The binding of the receptor with the ligand leads to its activation and internalisation.^49^ Although ERα was long considered to be a nuclear protein, it is now clear that it also works as a plasma membrane-localised receptor.^50–52^ The trafficking of plasma ERs has been challenging and the mechanisms regulating membrane ERα (mERα) levels have remained elusive.^53^ However, there is compelling evidence that activated membrane ERs can be internalised from the plasma membrane into cells.^54^ This mERα plays an important role in proliferation and other cellular functions.^55^ To investigate the role of the mERα on the internalisation of the ERα-AuNP nanotags, MCF-7 cells were pre-blocked with anti-ERα antibody (10 μg mL^−1^, 1 h), which binds to the ligand-binding site of the receptor on the outside of the cell. Therefore, blocking of the mERα by the free anti-ERα antibodies will occur and compete with the ERα antibodies attached to the nanotags. The results demonstrate that a suppression effect was observed in the cells pre-blocked with free anti-ERα antibody, since there was a much lower ERα-AuNP nanotag accumulation in MCF-7 cells pre-treated with unlabelled ERα antibody (Fig. 5A). Specifically, the 2D SERS maps of the pre-blocked MCF-7 cells showed that the SERS signal was obtained from the cell surface indicating the presence of the nanotags in the cell membrane and the absence of SERS signal in the interior of the cell (Fig. 5B). Additionally, a significant decrease in the SERS response was observed in the pre-blocked MCF-7 cells (from 1.8% ± 0.5 to 0.1% ± 0.2) confirming the importance of mERα for the uptake of ERα-AuNP nanotags (Fig. 5C). Moreover, the 3D Raman mapping showed that the SERS signal was observed throughout the cell only in the untreated samples (Fig. 5D and F). However, when the cells were pre-blocked with antibody, there was a significant reduction in the SERS signal through the *z*-plane (Fig. 5E and G). Specifically, the SERS signal seemed to come from the areas closer to the cell membrane than intracellularly, suggesting that the nanotags were adhering to the cell surface and not being internalised. The points that were used for the extraction of SERS spectra throughout the *z*-axis are shown in ESI, Fig. S13.† Moreover, ESI Fig. S14† represents the spectra from each 3 μm step size of the *z*-stack in the whole volume map. Coupling these 3D SERS results with the 2D Raman mapping, there is a clear indication that the membrane ERα appears to interact with the ERα-AuNP nanotags, suggesting their cellular uptake was an ERα receptor-mediated endocytosis process. Additionally, incubation of ERα-AuNPs with an ERα negative breast cancer cell line (triple negative MDA-MB-231 cells) confirmed that ERα mediated endocytosis is the distinct mechanism of cell uptake since MDA-MB-231 cells showed weaker SERS signal and lower nanotags accumulation compared to MCF-7 cells (ESI, Fig. S15†). This data is in agreement with the experiments performed at low temperature, where the low temperature slowed down the ligand–receptor binding rate which led to decreased internalisation of the ERα-AuNP nanotags. Hence, this was another clear indication that the SERS nanotags enter the MCF-7 cells through a receptor-mediated endocytosis mechanism. Studies have also shown that there are other ER-like membrane receptors such as ER-X and GPR30 that can be activated upon ligand binding.^56,57^ Therefore, future studies should be performed to investigate if the presence of these receptors have any possible contribution to the cellular uptake of the ERα-AuNP nanotags.

## Conclusions

In this study, we investigated the cellular uptake of gold nanoparticles functionalised with an anti-ERα antibody and BPE Raman reporter (ERα-AuNPs) using non-destructive 2D and 3D SERS imaging. 3D SERS cell mapping confirmed that ERα-AuNPs cellular uptake was temperature-dependent which excluded the passive transport mechanism as the uptake mechanism for their internalisation. 3D SERS images also confirmed that dynamin was responsible, at least in part, for the intracellular delivery of ERα-AuNPs in MCF-7 cells. Specifically, the 3D SERS suggested that ERα-AuNPs adhered to the plasma membrane due to the blocking of dynamin to form endocytic vesicles (ESI, Fig. S16A†). This mechanism suggests that ERα-AuNPs are internalised in an endosome. We assume that the nanoparticles are biologically active inside the cells since our current experimental work indicated that ERα-AuNPs show preference to ERα positive cells compared to the negative ones. Additionally, 3D SERS images showed that ERα-AuNPs entered MCF-7 cells using a receptor-mediated endocytosis after their binding to ERα located in the plasma membrane of the cells (ESI, Fig. S16B†). Hence, this study has provided an important biological insight into the intracellular uptake of nanotags by generating 3D SERS images of the entire cell volume, whilst maintaining the integrity of the cell. The novelty of this work also lies in the development of an accurate way for calculating the relative SERS response in MCF-7 cells under different conditions to give a qualitative indication of nanotag uptake per condition. In this way it was possible to acquire a relative assessment of ERα-AuNPs internalisation without the need for destructive, time consuming and expensive imaging, such as TEM. The study gives important insights into the uptake of functionalised SERS nanotags where a crucial fundamental understanding is required for their application in diagnostics and targeted drug delivery systems. Therefore, it is highlighted that SERS can be used as an excellent tool for investigating nanoparticle cellular uptake mechanisms for their potential use in cell imaging applications.

## Experimental

### Materials

Anti-estrogen receptor alpha antibody (ab16660) was purchased from Abcam (330 Cambridge Science Park, Cambridge, CB4 0FL, UK). Anti-mouse IgG HRP-linked antibody (7076S) and anti-rabbit IgG HRP-linked antibody (7074S) were purchased from Cell Signalling Technology (Hamilton House, Mabledon Place, London, WC1H 9BB, UK). Sodium tetrachloroaurate dihydrate, (*N*-(3-dimethylaminopropyl)-*N*′-ethylcarbodiimide hydrochloride) (EDC), *N*-hydroxysulfosuccinimide sodium salt (NHS), poly(ethylene glycol) 2-mercaptoethyl ether acetic acid (HS-PEG5000-COOH), dynasore hydrate, 1,2-bis(4-pyridyl)ethylene (BPE), 4-(2-hydroxyethyl)-1-piperazineethancesulfonic acid (HEPES), and 2-(*N*-morpholino)ethanesulfonic acid (MES) were obtained from Sigma-Aldrich Ltd (The Old Brickyard, New Road, Gillingham, Dorset, SP8 4XT, UK). LIVE/DEAD Viability/Cytotoxicity Assay Kit was purchased from ThermoFisher Scientific (3 Fountain Dr, Inchinnan, Renfrew PA4 9RF, UK). All glassware was cleaned in aqua regia (3 HCl : 1 HNO_3_).

### Nanoparticle synthesis and functionalisation

Citrate reduced gold nanoparticles (AuNPs) were synthesised according to the Turkevich, Stevenson and Hillier method.^35^ Briefly, sodium tetrachloroaurate dihydrate solution (10 mL, 15 mM) in 490 mL deionised water was boiled under continuous stirring. Sodium citrate tribasic dihydrate solution (7.5 mL, 26 mM) was then added. The mixture was boiled with stirring for about 1 h. The average diameter of the gold nanoparticles was measured to be approximately 40 nm by scanning electron microscopy. For SEM, silicon wafers were cleansed with methanol and allowed to air dry before adding 5 μL of AuNPs to again air dry. Environmental scanning electron microscope (ESEM) FEI Quanta 250 FEG-ESEM was used to image at an accelerating voltage of 30 kV. Image J (National Institute of Health (NIH)) with Fiji plug-in was used to measure the average diameter of the NPs. For the carbodiimide crosslinking conjugation, 74 μL of EDC solution (1 mg mL^−1^ in 10 mM MES, pH 6.0) was mixed with 40 μL of HS-PEG5000-COOH (12.5 μM in dH_2_O) followed by the addition of 217 μL of NHS (1 mg mL^−1^ in 10 mM MES, pH 6.0) and 20 μL of anti-ERα antibody (2.5 mg mL^−1^ in dH_2_O). The final solution was incubated in 669 μL of 10 mM HEPES buffer pH 7.0 on a shaker plate for 18 h at room temperature. 10 μL of 1,2-bis(4-pyridyl)ethylene (BPE) (0.1 μM) was added to bare AuNPs (0.03 nM, 990 μL) and the solution was incubated on the shaker plate for 30 min followed by centrifugation at 6000 rpm for 20 min. The solution of EDC–NHS-PEG5000-mAb was added dropwise to the pelleted BPE-AuNPs. The nanotags were incubated on a shaker plate for 3 h. Excess free protein was removed by centrifugation at 6000 rpm for 10 min and was used for protein concentration estimation analysis. The calculations for nanoparticle concentration were performed using UV-vis spectrometry and the Beer–Lambert law (*A* = *εcl*). The extinction coefficient (9.92 × 10^9^ M^−1^ cm^−1^) for 50 nm gold particles was used.^58^

### ERα-AuNP nanotags characterisation

Extinction spectra were measured using an Agilent Cary 60 UV-visible (UV-vis) spectrophotometer with Win UV scan V.2.00 software. The instrument was allowed to equilibrate to RT before using poly(methyl methacrylate) (PMMA) disposable plastic micro cuvettes with 500 μL sample volumes to scan wavelengths from 300–800 nm. Where required, samples were diluted to give extinction values of less than one to adhere to the Beer–Lambert law, to allow calculation of the concentration of AuNPs. Dynamic light scattering (DLS) and *z*-potential were measured using a Malvern Zetasizer Nano ZS with 800 μL of the sample in a PMMA disposable micro cuvette with Zetasizer μV and APS v.6.20 software. Polystyrene latex beads (40 nm) were used as a standard to validate the calibration of the system before running samples. Measurements were taken in triplicate. A scanning electron microscope (SEM) FEI Quanta 250 FEG-ESEM was used to image at an accelerating voltage of 30 kV and typically a spot size of 4 was selected, and an Everhart–Thornley detector collected secondary electrons. For the solution measurements of nanotags, SERS analysis was carried out on a Snowy Range CBEx 2.0 handheld Raman spectrometer (Snowy Range Instruments, Laramie WY USA) equipped with a 638 nm laser and maximum laser power of 40 mW. Samples were deposited in glass vials for interrogation. The sample volumes were 600 μL and spectra were collected using 100% laser power at the sample with a 0.05 s accumulation time. The software used to acquire spectra was Peak 1.1.112. Resulting spectra were baseline corrected in Matlab 2014b.^59^

### Cell culture and ERα-AuNP nanotags incubation

MCF-7 (ATCC® HTB-22™) human breast cancer cells were obtained from American Type Culture Collection (ATCC) (Queens Road, Teddington, Middlesex, TW11 0LY, UK). The cells were cultured in Rosewell Park Memorial Institute medium (RPMI 1640) supplemented with 1% penicillin/streptomycin (10 000 units per mL), 1% fungizone, and 10% heat-inactivated fetal bovine serum (FBS). Cells were incubated at 37 °C and 5% CO_2_ in a humidified incubator. Cells at a confluence of *ca.* 90% growing in a T175 flask were trypsinised and re-suspended in medium to give a concentration of *ca.* 1 × 10^6^ cells per mL. For fixed cell microscopy, the cells (1 × 10^6^ cells per mL) were seeded onto sterile 22 mm square glass coverslips with culture medium containing the ERα-AuNP SERS nanotags at 37 °C, 5% CO_2_ in a humidified incubator. Based on the experimental requirements, different concentrations of ERα-AuNP SERS nanotags (3 pM to 60 pM) and different incubation times (5 min to 120 min) were used. The coverslips were washed with PBS three times and fixed in 4% paraformaldehyde for 15 min. The fixed cells were washed with PBS and dH_2_O and left to air dry before mounting on a standard glass microscope slide for data collection. For temperature-dependent inhibition studies, MCF-7 cells (1 × 10^6^ cells per mL) were cultured in a 6-well dish for 24 h. The cells were then exposed to ERα-AuNPs for 2 h either at low temperature (4 °C) or at normal temperature (37 °C), washed with PBS three times and fixed with 4% paraformaldehyde for SERS imaging.

### Cell viability studies: live/dead cell staining assay

The assessment of cell toxicity was achieved using Invitrogen's LIVE/DEAD® Viability/Cytotoxicity Assay Kit for mammalian cells (#L3224, ThermoFisher Scientific). Briefly, the cells were plated at a density of 0.5 × 10^6^ cells per 35 mm Ibidi chamber and left to adhere overnight. The cells were exposed to PEG5000-AuNPs and ERα-AuNPs (60 pM) nanotags for 48 h at 37 °C. Prior to the assay, the cells were washed three times with PBS. A solution of Calcein AM (10 μL, 4 mM) and EthD-1 (20 μL, 2 mM) were diluted in 10 mL of PBS. The fluorescent staining solution (750 μL) was then added to the cells at 37 °C for 15 min before removing for imaging using a Leica Microsystems TCS SP8 with continuous wave visible lasers and a Leica DMi8 inverted microscope and DFC 7000T and TL LED cameras. The Leica Application Suite X V.3.1.5.16308 software was used to carry out live/dead studies using a Leica 63× magnification HC PL APO water objective with a 1.2 NA. Intensity and area of fluorescence was measured using Image J (National Institute of Health (NIH)) software with Fiji plug-in to measure the area of fluorescent stain.^60^

### Cell viability studies: trypan blue cell viability counts

MCF-7 cells were seeded in a 6-well plate (5 × 10^5^ cells per mL). After 24 h the cells were treated with BPE-AuNPs, PEG5000-AuNPs and ERα-AuNPs at a concentration of 60 pM for 48 h before counting. After 48 h the media was removed, cells were rinsed with PBS three times and 1 mL of trypsin was added to detach cells. Finally, 1 mL of complete RPMI medium was added to recover the cells for counting. The cells were diluted (1/5 dilution) before counting on a haemocytometer slide following addition of trypan blue viability dye. Each condition was conducted in triplicate. The number of live (non-blue) cells were recorded.

### Dynamin dependent endocytosis

MCF-7 cells (1 × 10^6^ cells per mL) were cultured in a 6-well dish for 24 h. The cells were then treated with 80 μM dynasore for 30 min at 37 °C before addition of 60 pM of ERα-AuNP nanotags for 2 h. The cells were washed with PBS three times and fixed with 4% paraformaldehyde for SERS imaging.

### Estrogen receptor mediated endocytosis

MCF-7 cells (1 × 10^6^ cells per mL) were cultured in a 6-well dish for 24 h. The cells were then exposed to free ERα (10 μg mL^−1^) for 1 h before the addition of 60 pM ERα-AuNP nanotags. The cells were incubated for 2 h. The coverslips were washed with PBS three times and fixed in 4% paraformaldehyde for 15 min. The fixed cells were washed with PBS and dH_2_O and left to be air dried and mounted to a standard glass microscope slide for data collection.

### Western blot experiments

Cells (1 × 10^6^ per mL) were plated in 10 cm diameter dishes with 10 mL RPMI and left for 24 h. The cells were washed with 1× ice cold PBS twice and were lysed with 200 μL ice cold RIPA buffer (#10017003, Thermo Fisher) containing protease and phosphatase inhibitors (#A32959, Pierce). Cell lysates (20 μL, 1 mg mL^−1^) were diluted with 5× SDS loading buffer, heated to 95 °C for 5 min and 20 μL of the denatured cell lysate loaded into a 12% gel (Mini Protean TGX stain free Pre-cast gels, #456-8085, Bio-Rad) and run at 140 V for 40 min. The gel was electrotransferred to a 0.2 μm nitrocellulose membrane (#170-4159, Bio-Rad) with the BioRad TransBlot Turbo Transfer System using the Midi gel 10 min transfer setting. The membrane was blocked with 5% BSA blocking buffer for 1 h at room temperature. After blocking, the membrane was incubated at 4 °C overnight whilst rocking with the appropriate primary antibody diluted in 5 mL 5% w/v BSA, 1× TBS, 0.1% Tween 20. The next day the membrane was washed three times for 5 min each with 15 mL of TBST buffer (1.5% Tween 20 in 1× TBS). The membrane was incubated with the appropriate secondary antibody in 10 mL of 5% BSA blocking buffer with gentle agitation for 1 h at room temperature and was washed three times for 5 min each with 15 mL of TBST buffer afterwards. Finally, for the detection of the proteins, the membrane was incubated with 1 : 1 of Pierce™ ECL western blotting substrate (#32106, Thermo Fisher) for 1 min. A Bio-Rad ChemiDoc MP Imaging System-Universal Hood III with Image Lab V.4.1 software was used to image and quantify the protein levels on the membrane.

### Raman cell mapping

The intracellular uptake of the nanotags was examined using Raman cell mapping. Both 2D and 3D SERS mapping was performed in fixed cells to provide a “snapshot” of the nature and distribution of molecules nanoparticles within the cells. Cell fixation minimised the chances of cell movement and sample degradation during the analysis. Finally, the initial stages of dynamin mediated endocytosis occurring on a shorter timescale than SERS measurement (in approximately 1 min) than a 3D SERS measurement (approximately 2 h). Therefore, fixed cells are preferred to avoid blurring the images obtained from the cell phenomenon. A Renishaw InVia Raman confocal microscope with laser power of 1.2 mW (10% power) at the sample, from a HeNe 633 nm excitation source with a 0.1 s acquisition time per point, and a 1200 L mm^−1^ grating in high confocality mode was used to create initial depth profiles and establish the focal plane of cells in correlation with the white light images. Subsequently, 3D SERS maps were collected in edge Streamline HR high confocality mode at 1 μm resolution in the *X* and *Y* directions and 3 μm between *Z*-stacks. 3D SERS mapping was performed in cells with similar height characteristics were selected for the mapping experiments to have a comparable approach. Therefore, cells with similar height characteristics (*Z* = 15) were selected to perform the comparable study. Before each map, MCF-7 cells were analysed under the microscope and the *Z* = 0 was set accordingly. A 50× magnification NIR APO Nikon water immersion objective with a 0.75 NA was used on the samples. Windows-based Raman Environment (WiRE™ – Renishaw plc) 4.4 software package was used to pre-process the data for cosmic ray removal and baseline subtraction. The image was generated using direct classical least square analysis (DCLS) based on BPE reference spectrum. Therefore, the false colour was generated only when there was a good spectral fit between the reference and the collected spectra. All SERS experiments contained *n* = 10 biological replicates and experiments were in triplicate.

### Calculation of relative SERS response value in MCF-7 cells

The SERS response in cells was evaluated using Fiji image processing package^60^ by taking into consideration the pixel numbers of the nanotags and cell area after the SERS mapping. This was an estimation of the SERS response per cell and not a quantification of the total number of nanotags in the cells. Using this approach, we were able to estimate the SERS signal per cell area, quantify the pixels that corresponded this signal, identify the localisation of the nanotags, and allow the comparison between different samples. Before the analysis of the ERα-AuNP nanotag uptake, all the cells were scanned using 3D SERS to verify their intracellular distribution. The instrumental conditions were the same as those used for the Raman cell mapping and the spectra processed in the same manner using WiRE 4.4 software. Again, the images were generated using DCLS based on the Raman reporter reference spectrum. DCLS was used to fit the unknown data (collected during cell mapping) to a linear combination of the specified component spectrum (Raman reporter reference spectrum). If there was a good spectral fit between the Raman reporter reference and the collected spectra a gradient red false, colour was assigned. Associated with each false colour image was a look up table (LUT). The minimum and maximum values of the LUT indicates the degree of spectral fit. The minimum value of LUT was set to 0.4, which showed a good overlapping of the BPE reference spectrum with the collecting spectra. The gradient red false colour was then converted to monochromatic red colour, without affecting the intracellular SERS signal, using the WiRE 4.4 software package. The cellular area was selected by masking everything outside of it using Fiji image processing. The image was then colour split to the monochromatic red channel, where only the red pixels were present. A 200-threshold was set to count only the pixels that correspond to the nanotags and not to any cellular component (ESI, Fig. S17†). Finally, the percentage of the red pixel area (corresponding to SERS response) *versus* the full cell area was calculated (ESI, Fig. S18†). Before each map, the cells were analysed under the microscope, and the *z* = 0 was set accordingly. The mapping was performed with 3 μm micrometer step size. Additional information can be found in the Experimental part of the manuscript under the “Raman cell mapping” section.

### Statistical analysis

Statistical analysis was carried out on GraphPad Prism 8.1.2 (GraphPad Software, Inc., San Diego, CA). The Student's *t*-test was used for comparison of two variables and one-way analysis of variance (ANOVA) for comparison of three or more groups. Differences between groups were considered to be significant at a *P* value of <0.05.

## Conflicts of interest

There are no conflicts to declare.

## Supplementary Material

SC-011-D0SC01255F-s001
