# Supplementary material for: Investigation of cellular uptake mechanism of functionalised gold nanoparticles into breast cancer using SERS
Source: Chem Sci. 2020 May 27;11(22):5819–29. doi: 10.1039/d0sc01255f (PMC8159335; doi:10.1039/d0sc01255f)
Supplement: SC-011-D0SC01255F-s001 [file SC-011-D0SC01255F-s001.pdf]

Electronic Supplementary Material (ESI) for Chemical Science.
This journal is © The Royal Society of Chemistry 2020

Supplementary Information

Received 00th January 20xx

Accepted 00th January 20xx

DOI: 10.15129/73c3f6a3-ef37-4637-b775-53faf379f65e

Investigation of Cellular Uptake Mechanism of Functionalised Gold Nanoparticles into Breast Cancer Using SERS

Anastasia Kapara[a,b], Valerie Brunton[b], Duncan Graham[a], Karen Faulds[a*]

*karen.faulds@strath.ac.uk

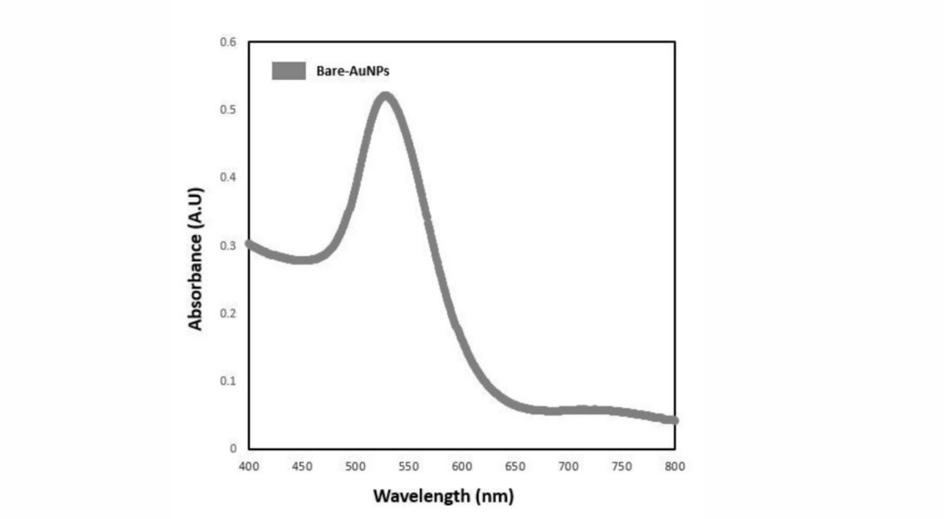

	Peak 1 Mean (d.nm)	Peak 2 Mean (d.nm)	Peak 1 %	Peak 2 %	Mean z-average	PdI
	53.35	4.38	88.4	11.6	-32.57	0.367
	52.30	4.58	88.3	11.7	-30.25	0.482
	51.81	4.67	84.6	15.4	-31.75	0.366
**Average**	**52.49**	**4.54**	**87.1**	**12.9**	**-31.52**	**0.405**
**STDEV**	**0.79**	**0.15**	**2.2**	**2.2**	**-1.18**	**0.067**

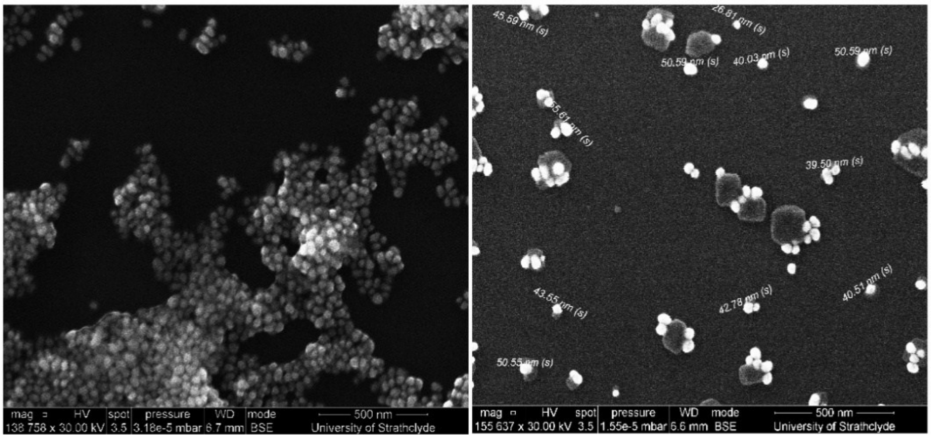

SI Figure S1. Characterisation of bare 40 nm citrate reduced AuNPs in dH20. (A) Extinction spectra of AuNPs revealed a sharp and narrow plasmon band at 529 nm indicating colloid monodispersity. (B) Differential light scanning analysis (DLS) of AuNPs showing that the average size of AuNPs was 52.5 ± 0.79 nm with a narrow size distribution. (C) Scanning electron microscopy (SEM) of AuNPs agreed with the DLS measurements and confirmed that AuNPs had a spherical shape with a diameter distribution ranging from 40 to 50 nm. The SEM also shows salt crystals as there are probably salt residues in the solution occurred during nanoparticle synthesis.

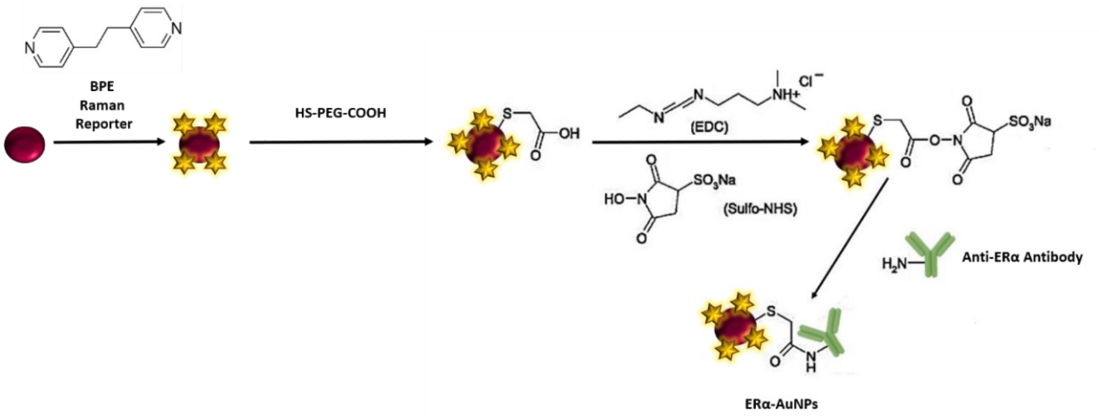

SI Figure S2. Schematic of ERα-AuNPs conjugation. AuNPs are first functionalised with BPE Raman reporter to create a monolayer. BPE has been used routinely as a Raman reporter in SERS bioanalysis since it is a non-resonant molecule, with low fluorescent background, resulting in high Raman signal. Carbodiimide crosslinking chemistry creates an amide bond between the HS-PEG5000-COOH and the amine of an antibody via the addition of EDC and sulfo-NHS. 1,2-Di(4-pyridyl)ethylene Raman reporter binds to AuNPs surface via its pyridyl nitrogens.[36,37]

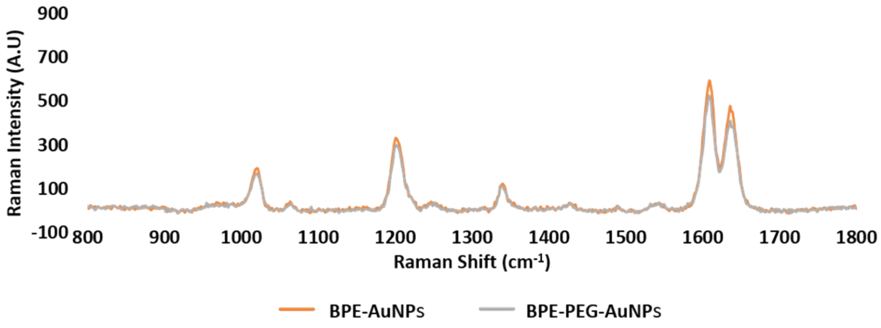

SI Figure S3. SERS spectrum of BPE-AuNPs and BPE-PEG-AuNPs in an aqueous solution. No displacement of the Raman reporter from the AuNPs surface upon addition of PEG was observed. The SERS analysis was carried out using a Snowy Range CBEx 2.0 handheld Raman spectrometer equipped with a 638 nm laser with a maximum laser power of 40 mW. The spectrum was collected using 100% laser power with 0.05 s accumulation time. The software used to acquire spectra was Peak 1.1.112

 This journal is © The Royal Society of Chemistry 20xx

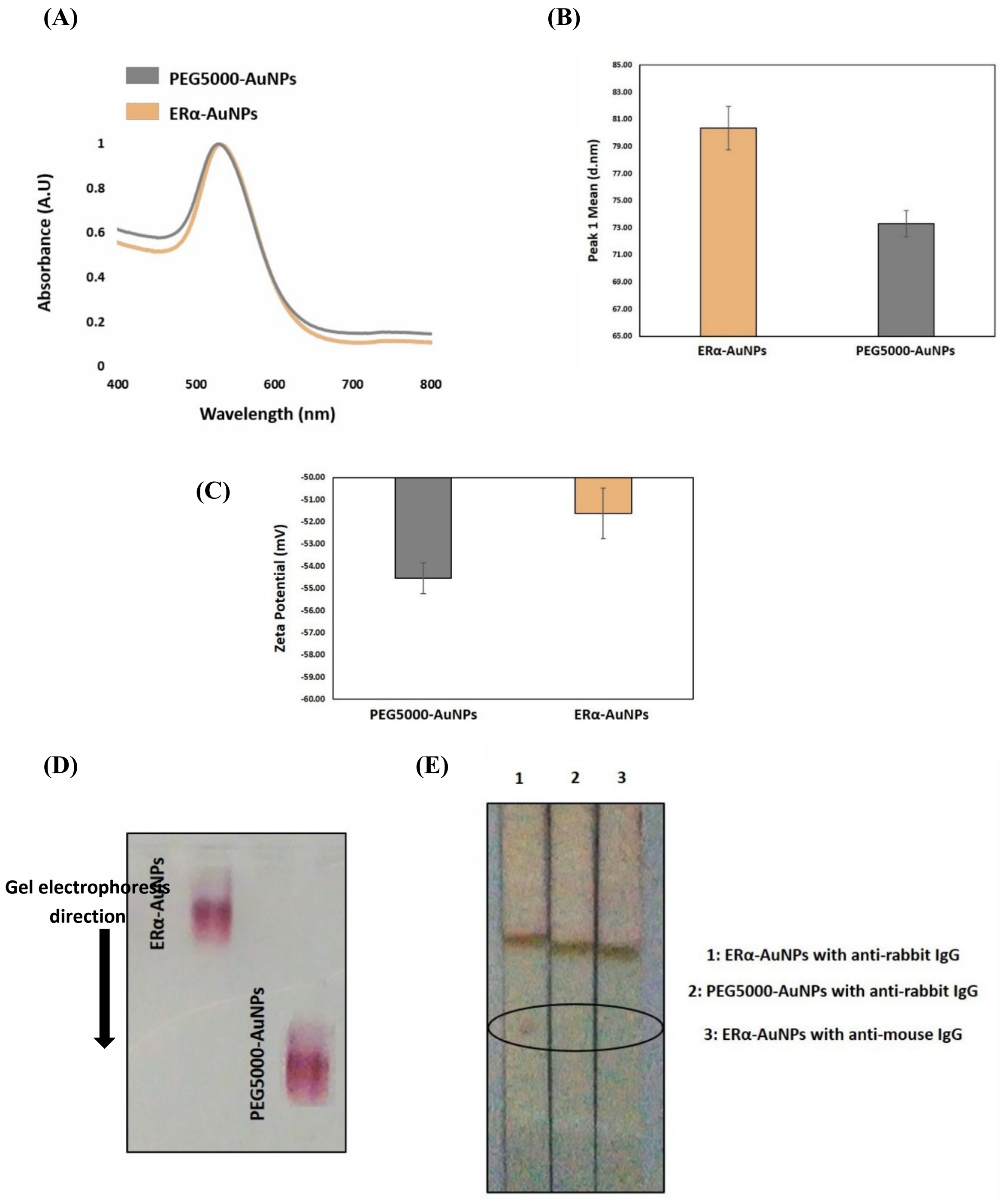

This journal is © The Royal Society of Chemistry 20xx

Please do not adjust margins

SI Figure S4. Characterisation of ERα-AuNPs after their functionalisation (A) Extinction spectra of BPE-AuNPs (blue), PEG5000-AuNPs (grey) and ERα-AuNPs (orange) nanotags showing that there was a shift in the wavelength when the antibody was added to the surface of AuNPs (from 529 to 533 nm). (B) Differential light scanning analysis (DLS) of PEG5000-AuNPs (grey) and ERα-AuNPs (orange) nanotags confirmed the successful functionalisation of the anti-ERα antibody as the hydrodynamic diameter of AuNPs increased and became more positive as each layer was added. The ERα-AuNPs were 80 ± 1.6 d.nm in comparison to the PEG5000-AuNPs (73 ± 1.0 d.nm) at pH 7.0. (C) Z-potential of PEG5000-AuNPs (grey) and ERα-AuNPs (orange) nanotags showing the increase of the zeta potential values (from -56 ± 0.7 mV to -52 ± 1.1 mV). This was a further verification of the anti-ERα antibody attachment to the AuNPs surface since the antibody carried a slightly positive charge at pH 7.0 (isoelectric point of anti-ERα antibody: 8.3) that increased the charge of the AuNPs. (D) Agarose gel after electrophoresis showing the distance travelled by PEG5000-AuNPs and ERα-AuNP nanotags. Gel electrophoresis is a method of separation and analysis, based on the size and charge of the samples being analysed. Here, gel electrophoresis confirmed the PEG5000-AuNPs travelled further than the ERα-AuNPs suggesting that the nanotags were of different size and/or charge and successful antibody functionalisation. (E) Lateral flow immunosorbent assay strips showing the spot from ERα-AuNPs onto the detection zone of the nitrocellulose strip. The spot was present only for samples with the matching secondary IgG antibody for ERα applied (anti-rabbit). There was no detected spot when the nanotags were applied to a lateral flow that contained a non-specific secondary IgG antibody (anti-mouse) or when PEG5000-AuNPs was tested with the anti-rabbit IgG confirming the successful binding of the anti-ERα antibody on the AuNPs surface.

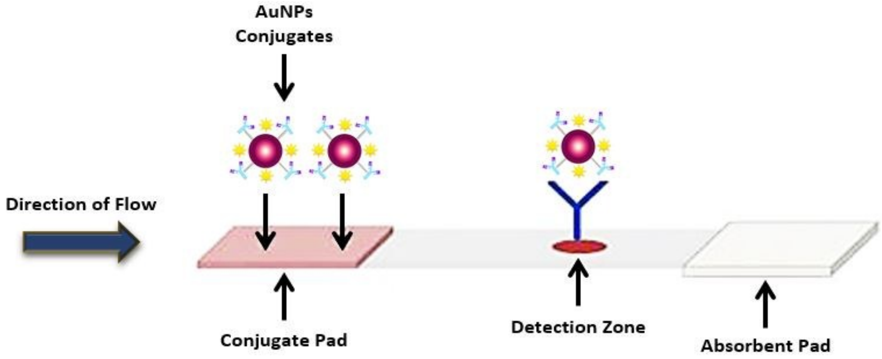

SI Figure S5. Schematic of lateral flow immunosorbent assay (LFA). For the lateral flow immunosorbent assay, the strips contained a sample pad dipped in the eluent (HEPES buffer), a conjugate pad to which the nanotags were applied, a nitrocellulose strip, where the antigen is immobilised and, finally, an absorbent pad which collects the excess eluent and nanotags Afterwards, the strip is placed in HEPES buffer which flows through the conjugate pad allowing the conjugate to join the flow on the strip. When the AuNP nanotags flow over the secondary antibody, they bind to it which immobilise the nanotags on the detection spot.

This journal is © The Royal Society of Chemistry 20xx

Please do not adjust margins

Please do not adjust margins

Sample	Absorbance	Concentration (mg/mL)	Concentration (µg/mL)
Std 1	0.1128	0.25	250
Std 2	0.1316	0.5	500
Std 3	0.1648	1	1000
Std 4	0.1950	1.5	1500
Std 5	0.2144	2	2000
Sample 1	0.1079	0.0196	19.6
Sample 2	0.1071	0.0233	23.3
Average	0.1070	0.0214	21.4
STDEV	0.0001	0.0026	2.6

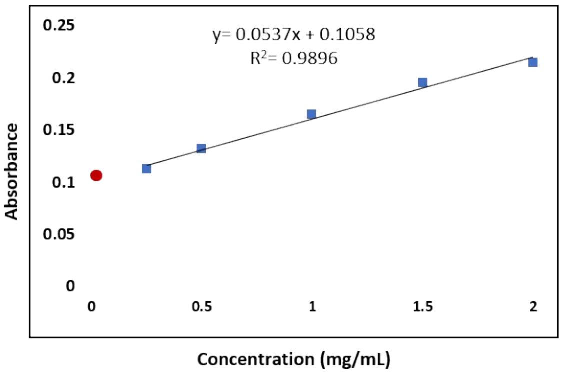

Added Antibody Concentration (µg/mL)	Supernatant Concentration (µg/mL)*	Adsorbed Antibody Concentration (µg/mL)**	Antibody Molecules per AuNPs (Ab/AuNPs)***
50	21.41 ± 2.63	28.58 ± 2.63	63.81 ± 5.88

SI Figure S6. Raw Data and calculations to determine the ERα antibody loading onto AuNPs using the bicinchoninic acid assay (BCA). *Concentration of remaining antibody in the supernatant calculated from the calibration curve. Absorbance was corrected by subtracting the absorbance of the PEG5000-AuNPs control sample (0 µg/mL antibody) per Bio-Rad protocol. The red dots in the BCA assay represent ERα-AuNPs sample. **Amount of antibody absorbed onto AuNPs presented as the concentration and calculated as the difference in the antibody added and antibody remaining in the supernatant. *** The average number of antibody molecules adsorbed onto each AuNPs was calculated by dividing the concentration of adsorbed antibody (converted to 178.65 nM using antibody MW of 160,000 g/mL) by the concentration of AuNPs (Initial concentration was 0.028 nM and AuNPs were centrifuged and concentrated to 2.8 nM).

Please do not adjust margins

Please do not adjust margins

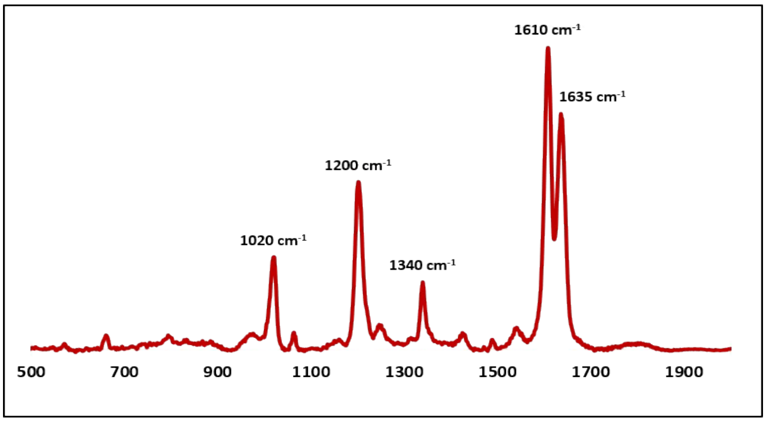

SI Figure S7. SERS spectrum of BPE Raman reporters adsorbed on 40 nm AuNPs in an aqueous solution at 10^{-7} M concentration. The SERS analysis was carried out using a Snowy Range CBEx 2.0 handheld Raman spectrometer equipped with a 638 nm laser with a maximum laser power of 40 mW. The spectrum was collected using 100% laser power with 0.05 s accumulation time. The software used to acquire spectra was Peak 1.1.112.

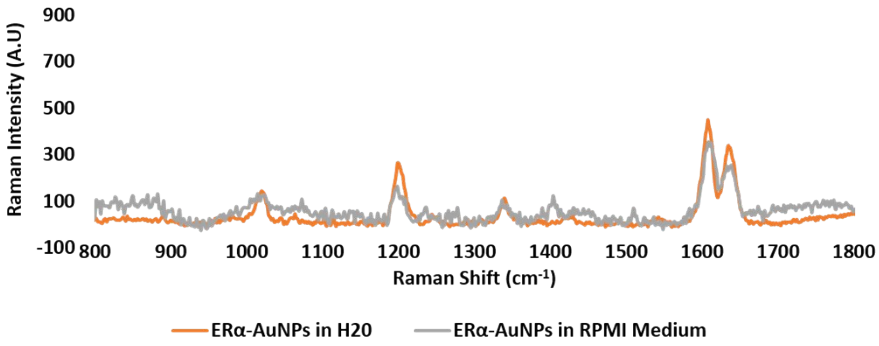

SI Figure S8. SERS intensity of ERα-AuNPs prepared in H20 or RPMI media. SERS analysis was carried out on Snowy Range CBEx 2.0 handheld Raman spectrometer (Snowy Range Instruments, Laramie WY USA equipped with a 638 nm laser with a maximum laser power of 40 mW).Samples were deposited in glass vials for interrogation. Spectra were collected using 10% laser power at the sample with a 1.0 s accumulation time. The software used to acquire spectra was Peak 1.1.112. Resulting spectra were baseline corrected in Matlab 2014b.

This journal is © The Royal Society of Chemistry 20xx

Please do not adjust margins

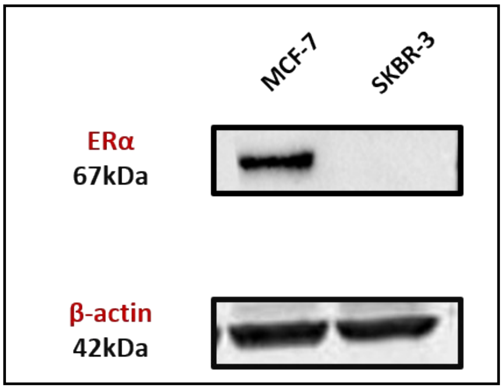

SI Figure S9. ERα expression in breast cancer cell lines. Cell lysates were prepared from breast cancer lines and western blot analysis carried out using an antibody to ERα, b-actin was used as a loading control.

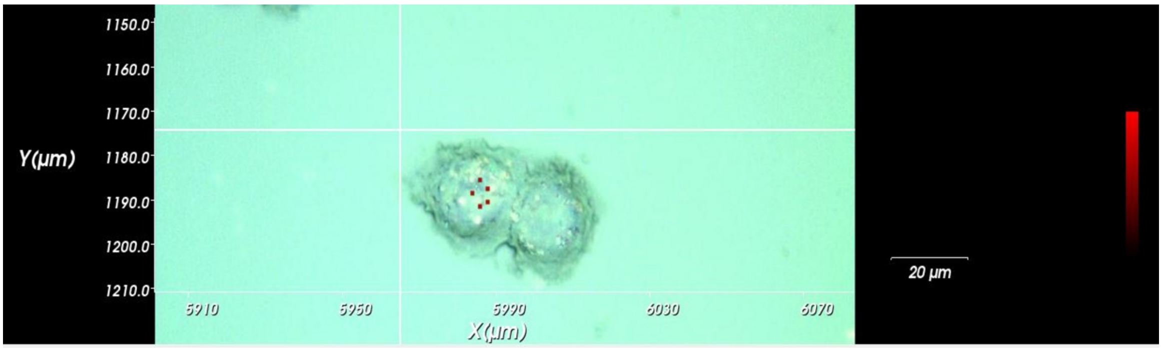

SI Figure S10. Top view of False colour SERS map for MCF-7 cells incubated with only ERα-AuNP nanotags (60 pM, 2 h). The images were generated using a Renishaw InVia Raman microscope with 50× magnification NIR APO Nikon water immersion objective with a 0.75 NA and 1.2 mW laser power (10% power) from a HeNe 633 nm excitation source with step size y,x 1.0 μm, 0.1s acquisition time and a 1200 L mm^{-1} grating in high confocality mode. Scale bar= 20 μm.

(A) (B)

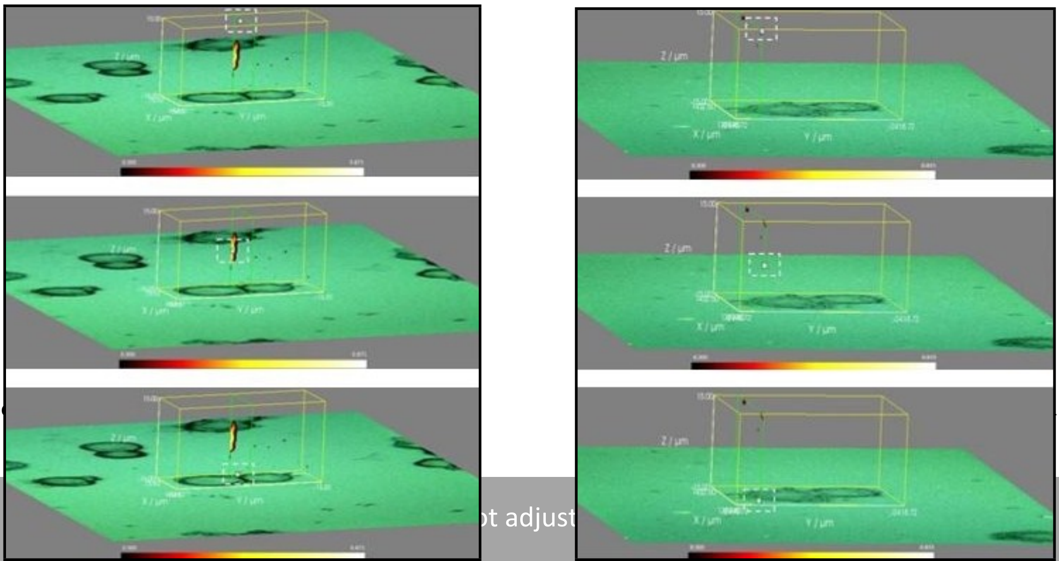

Please do not adjust margins

SI Figure S11. 3D Raman mapping results and SERS spectra detected into different z-axis points. The white dashed box depicting the z-axis of the image from where the SERS signal was extracted. (A) Untreated MCF-7 cells: as the z-axis moves from top to bottom of the cell, the BPE labelled ERα-AuNPs signal appeared throughout the MCF-7 cell. (B) When the cells were treated with ERα-AuNP nanotags (60 pM, 2 h) and dynasore (80 µM, 30 min). 3D SERS images were generating using a Renishaw InVia Raman microscope with 50× magnification NIR APO Nikon water immersion objective with a 1.0 NA and 1.2 mW laser power (10% power) from a HeNe 633 nm excitation source with step size y,x=1.0 µm and z= 3.0 µm, 0.1 s acquisition time and a 1200 L mm^{-1} grating in high confocality mode. The minimum and maximum look up table thresholds were set to exclude any poorly correlating or noisy spectra (min= 0.4). Coupling the data with the 2D Raman mapping, there is an indication that ERα-AuNPs did not enter the MCF-7 cells when dynasore was present.

(A)

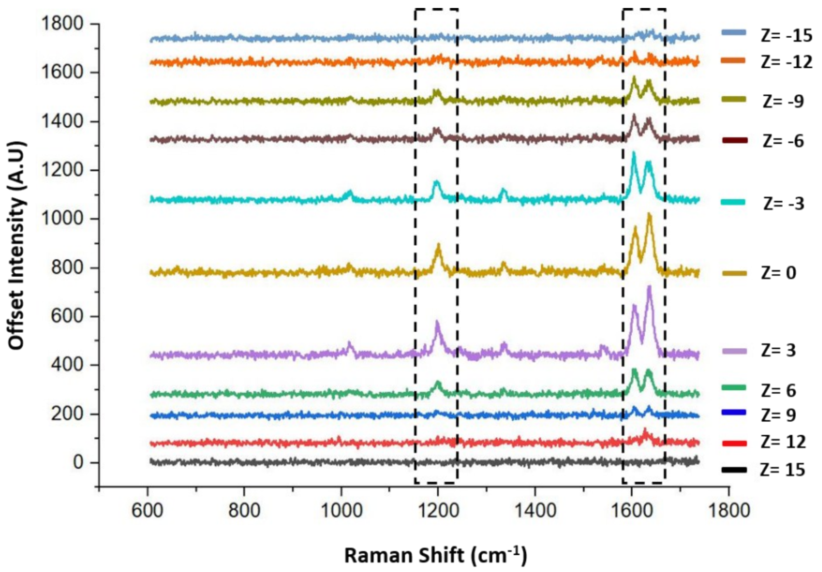

(B)

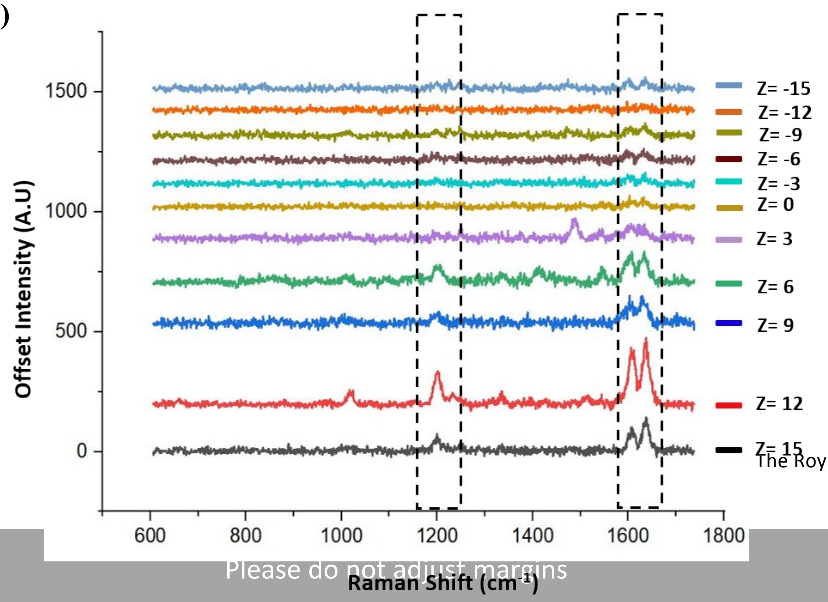

Please do not adjust margins

Please do not adjust margins

SI Figure S12. SERS spectra detection from all z-steps in the volume map. **(A)** Untreated MCF-7 cells **(B)** MCF-7 cells treated with ERα-AuNP nanotags (60 pM, 2 h) and dynasore (80 M, 30 min). 3D SERS images were generating using a Renishaw InVia Raman microscope with 50× magnification NIR APO Nikon water immersion objective with a 1.0 NA and 1.2 mW laser power (10% power) from a HeNe 633 nm excitation source with step size y,x=1.0 µm and z= 3.0 µm, 0.1 s acquisition time and a 1200 L mm^{-1} grating in high confocality mode.

(A) **(B)**

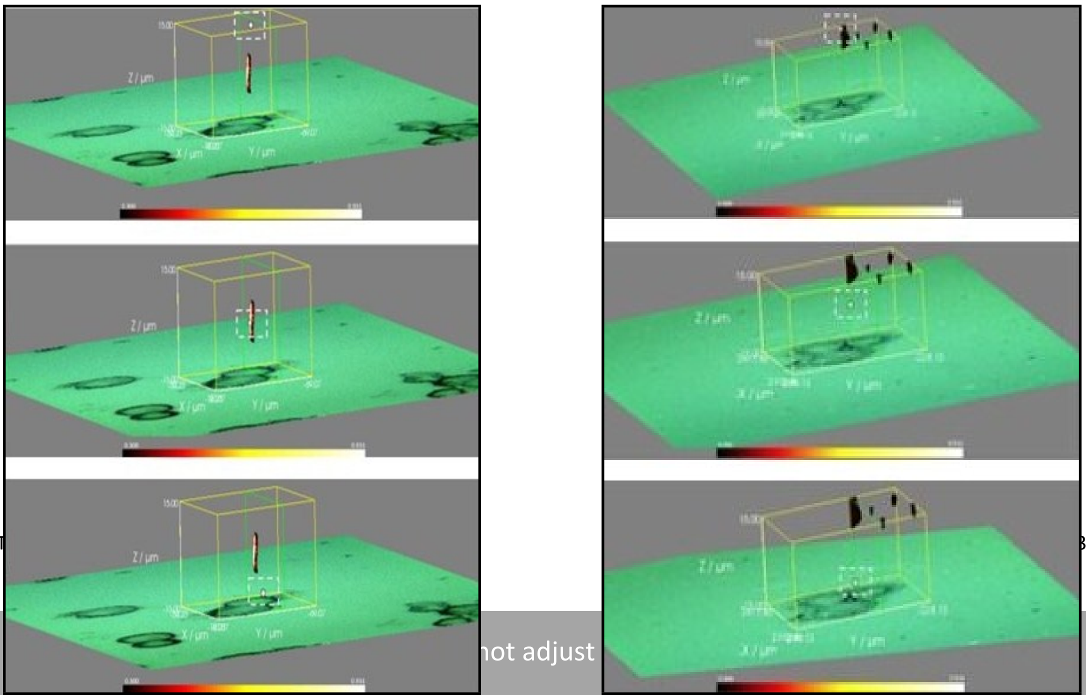

[illegible]not adjust[illegible]

Please do not adjust margins

SI Figure S13. 3D Raman mapping results and SERS spectra detected from different z-axis points. The white dashed box depicting the z-axis of the image from where the SERS signal was extracted. (A) Untreated MCF-7 cells: as the z-axis moves from top to bottom of the cell, the BPE labelled ERα-AuNPs signal appeared throughout the MCF-7 cell. (B) When the cells were preblocked with free anti-ERα antibody (10 µg/mL, 1 h) there was a significant reduction in the SERS signal throughout the z-axis. 3D SERS images were generating using a Renishaw InVia Raman microscope with 50× magnification NIR APO Nikon water immersion objective with a 1.0 NA and 1.2 mW laser power (10% power) from a HeNe 633 nm excitation source with step size y,x=1.0 µm and z= 3.0 µm, 0.1 s acquisition time and a 1200 L mm^{-1} grating in high confocality mode. The minimum and maximum look up table thresholds were set to exclude any poorly correlating or noisy spectra (min= 0.4). Coupling the data with the 2D Raman mapping, there is an indication that ERα-AuNPs did not enter the MCF-7 cells when they were preblocked with the anti-ERα antibody.

(A)

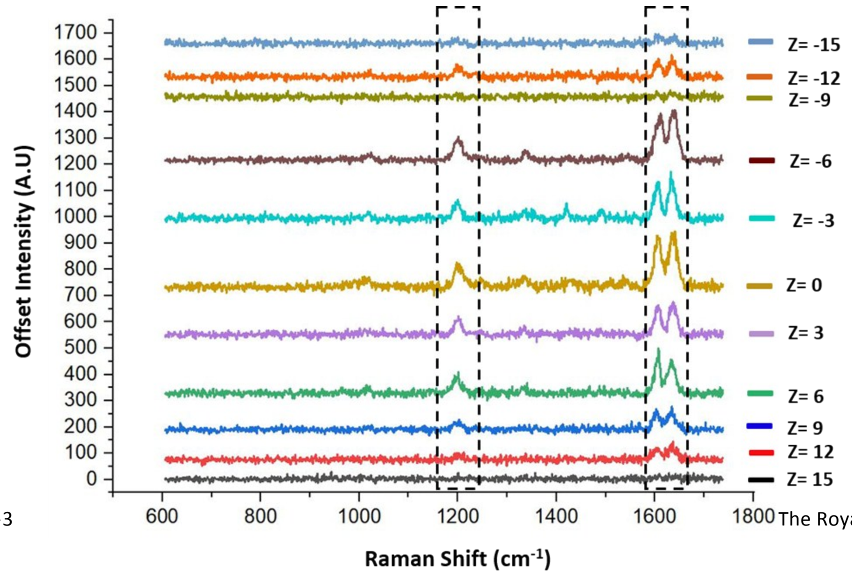

Please do not adjust margins

(B)

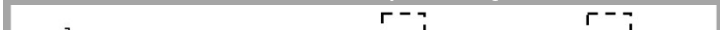

Please do not adjust margins

SI Figure S14. SERS spectra detection from all z-steps in the volume map. (A) Untreated MCF-7 cells (B) MCF-7 cells treated with ERα-AuNP nanotags (60 pM, 2 h) and free anti-ERα antibody (10 µg/mL, 1 h). 3D SERS images were generating using a Renishaw InVia Raman microscope with 50× magnification NIR APO Nikon water immersion objective with a 1.0 NA and 1.2 mW laser power (10% power) from a HeNe 633 nm excitation source with step size y,x=1.0 µm and z= 3.0 µm, 0.1 s acquisition time and a 1200 L mm^{-1} grating in high confocality mode.

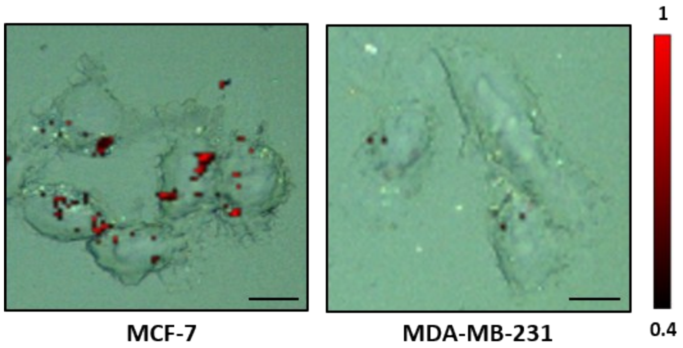

SI Figure S15. SERS map of MCF-7 and MDA-MB-231 cells with 60 pM ERα-AuNPs nanotags incubated for 2 h at 37 ^{o}C. Images were generated with a 50× magnification NIR APO Nikon water

This journal is © The Royal Society of Chemistry 20xx

Please do not adjust margins

immersion objective with a 1.0 NA, laser power of 1.2 mW (10% power) at the sample, from a HeNe 633 nm excitation source with a 0.1 s acquisition time per point, and a 1200 L mm^{-1} grating in high confocality mode. The false coloured images represent the ERα-PEG5000-AuNPs were generated using the Windows-based Raman Environment (WiRE™ - Renishaw plc) 4.4 software package on a Renishaw InVia microspectrometer and direct classical least square analysis (DCLS) based on a BPE Raman reporter reference spectrum. The minimum and maximum look up table (LUT) thresholds were set to exclude any poorly correlating or noisy spectra, (min= 0.4, max= 1). Scale bars= 10 µm.

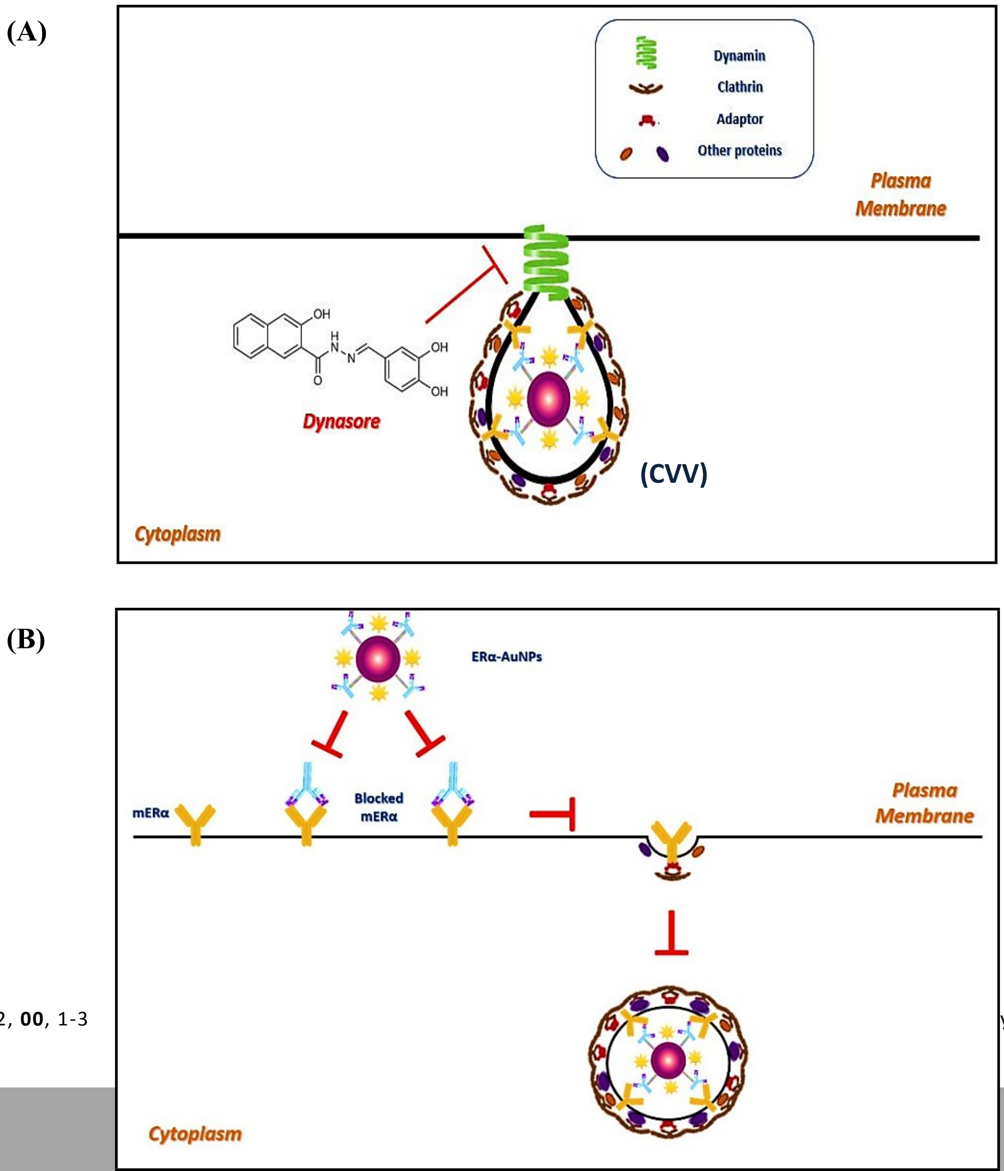

SI Figure S16. ERα-AuNPs are using dynamin and mERα for their internalisation in MCF-7 cells. (A) Schematic representing the action of dynasore in mammalian cells. Clathrin coated vesicle (CCV) is formed, and cargo-specific adaptors are selected after the incubation of ERα-AuNP nanotags. Dynamin is recruited to the neck of the forming vesicle to induce plasma membrane scission. Dynasore inhibits the GTPase activity of dynamin, blocking constriction and fission and, therefore, nanotags are trapped into the submembrane regions. (B) Schematic diagram of blocking mERα in MCF-7 cells with the free anti-ERα antibody. Free anti-ERα antibodies compete with anti-ERα antibodies attached on nanotags, resulting in blocking of ERα-AuNPs internalization via mERα-mediated endocytosis. CCV is, therefore, not formed. In normal conditions, for their internalisation, ERα-AuNPs first bind to mERα, forming a mERα-ERα-AuNPs complex. The complex binds the coat proteins, and CCV assembly begins. The CCV either grows to form a vesicle and the ERα-AuNP is then internalized.

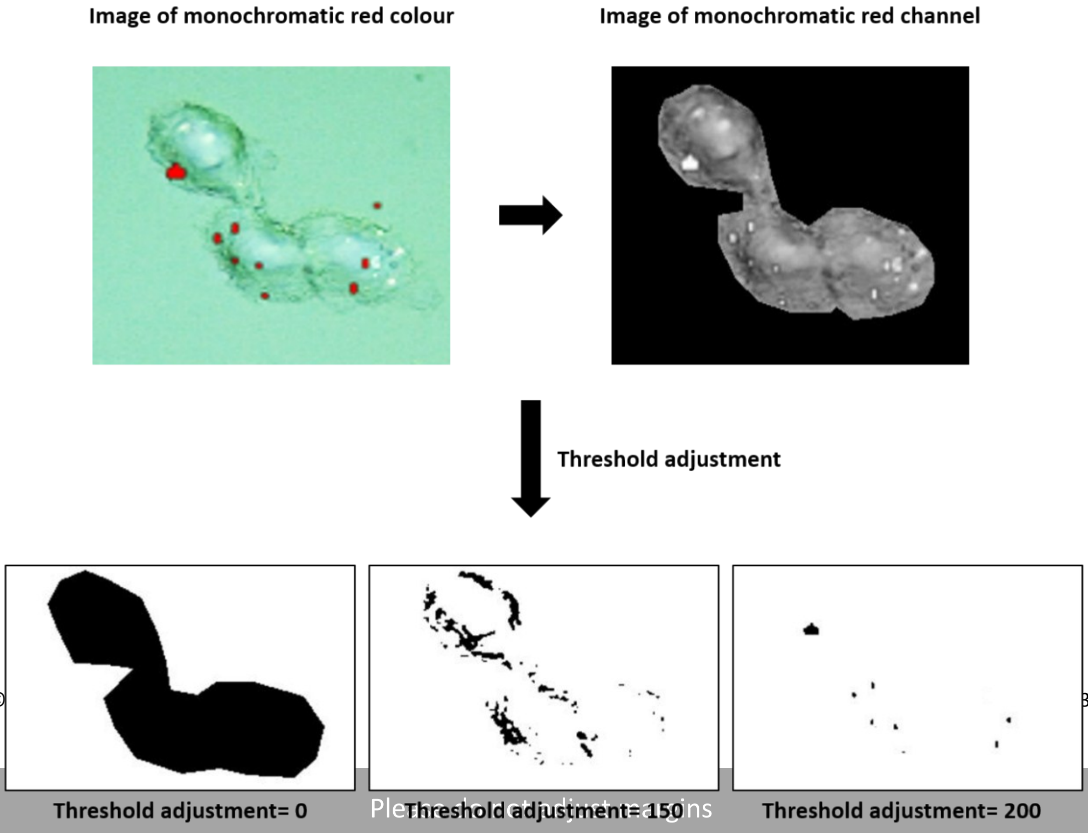

Please do not adjust margins

SI Figure S17. Schematic representing the pre- and post-threshold removal. The threshold value of 200 was selected for the calculation of relative SERS response since it overlaps with ERα-AuNPs signal from SERS cell mapping.

This journal is © The Royal Society of Chemistry 20xx

Please do not adjust margins

Please do not adjust margins

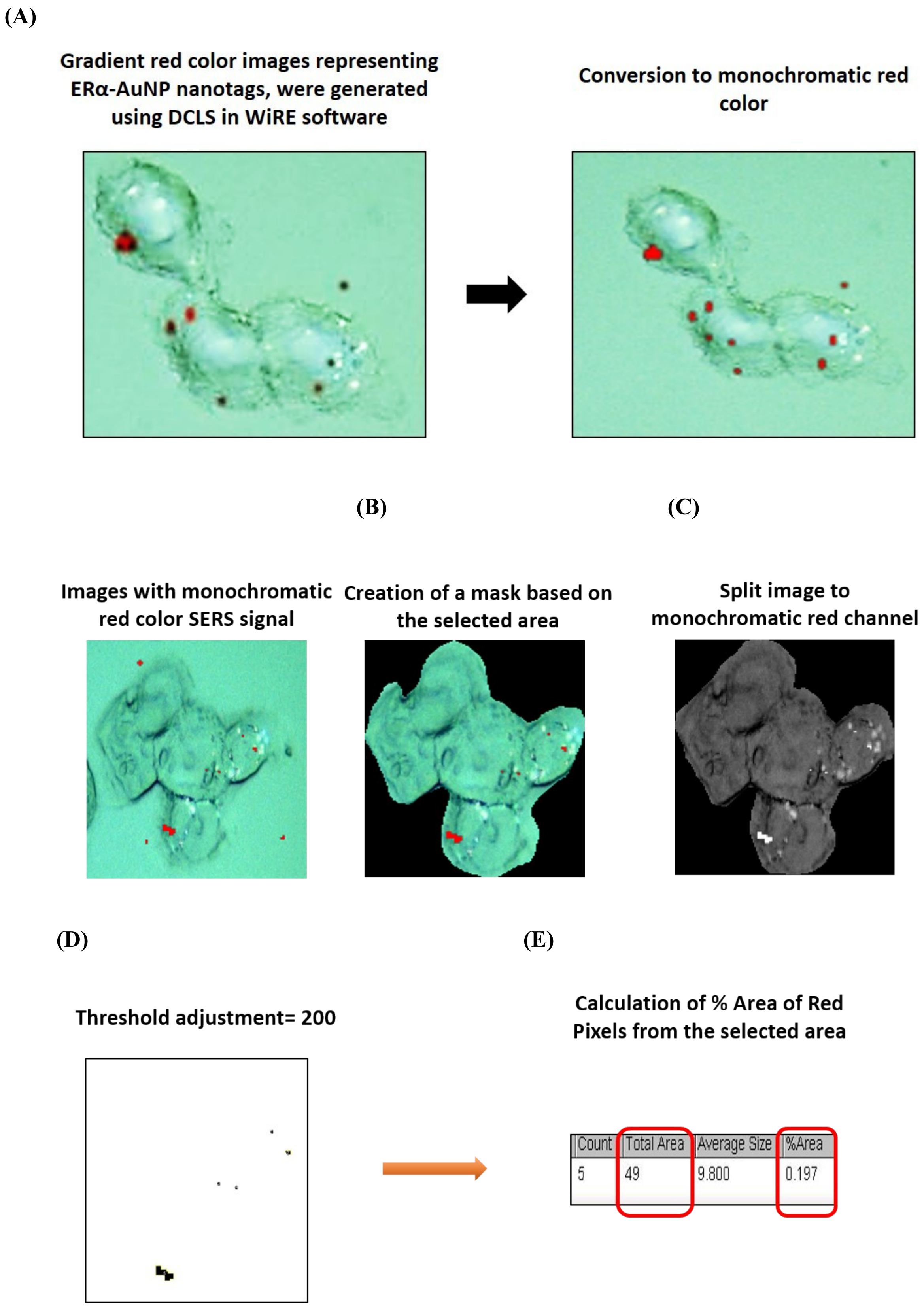

Please do not adjust margins

Please do not adjust margins

SI Figure S18. Schematic representing the step carried out for the calculation of relative SERS response in MCF-7 cells using WiRE™ - Renishaw plc 4.4 and Fiji image processing package. (A) After the generation of false colour images using DCLS from WiRE™ - Renishaw plc 4.4 software, the gradient red false colour was converted to monochromatic red colour, without affecting the intracellular SERS signal. The images with the monochromatic red signal were then analysed using Fiji. (B) The cellular area was then selected by masking everything outside it. (C) Afterwards, the image was colour split to the monochromatic red channel where only the red pixels were represented as bright white spots. (D) Only the red pixels that were above 200-threshold were extracted for the calculations. (E) Finally, the percentage of the red pixel area (corresponding to SERS response) versus the full cell area was calculated. 10 cells per sample group were analysed. MCF-7 cells were fixed in 4% paraformaldehyde, therefore, cells were not moving during the scanning. The percentage of SERS response was plotted and analysed statistically using one-way analysis of variance (ANOVA).

This journal is © The Royal Society of Chemistry 20xx

Please do not adjust margins
